# High-Pressure Supercritical CO_2_ Extracts of *Ganoderma lucidum* Fruiting Body and Their Anti-hepatoma Effect Associated With the Ras/Raf/MEK/ERK Signaling Pathway

**DOI:** 10.3389/fphar.2020.602702

**Published:** 2020-12-14

**Authors:** Liping Zhu, Min Wu, Peng Li, Yanfei Zhou, Jinyi Zhong, Zhiqiang Zhang, Ye Li, Weixi Yao, Jianhua Xu

**Affiliations:** ^1^ Department of Pharmacology, Department of Natural Medicine, School of Pharmacy, Fujian Provincial Key Laboratory of Natural Medicine Pharmacology, Fujian Medical University, Fuzhou, China; ^2^ Fujian Xianzhilou Biological and Technology Co., Ltd., Fuzhou, China

**Keywords:** supercritical CO_2_ extract, *Ganoderma lucidum*, triterpenoid, antitumor, hepatoma

## Abstract

As a noted medicinal mushroom, *Ganoderma lucidum* (*G. lucidum*) has been reported to have a number of pharmacological effects such as anti-tumor and liver protection. Compared with the common ethanol reflux method, supercritical CO_2_ extraction has obvious advantages in obtaining antitumor extracts from *G. lucidum* fruiting body such as short extraction time, low temperature and no solvent residue. However, Using high-pressure supercritical CO_2_ without entrainer to obtain the antitumor extracts from *G. lucidum* and studying their anti-hepatoma effect have not been reported. In this study, high-pressure supercritical CO_2_ extracts obtained under 65, 85, and 105 MPa pressure named as G65, G85, G105 respectively and ethanol reflux extract (GLE) were used to investigate their anti-hepatoma activity and the underlying molecular mechanism. The total triterpenoid content of G85 was significantly higher than that of G65 and GLE, but did not differ significantly from that of G105 by UV and high-performance liquid chromatography. GLE, G65, and G85 could inhibit cell proliferation, arrest cell cycle in G2/M phase, and induce apoptosis in two liver cancer cell lines (QGY7703 and SK-Hep1), of which G85 had the strongest effect. The results showed that the potency of their cytotoxicity of the high-pressure supercritical CO_2_ extracts on human hepatoma carcinoma cells *in vitro* was consistent with their total triterpenoid content. G85 exhibited significant anti-hepatoma effect with low toxicity *In vivo*. Further mechanistic investigation revealed that the anti-tumor effect of these extracts was associated with their inhibition of Ras/Raf/MEK/ERK signaling pathway. Our findings suggest that the high-pressure supercritical CO_2_ extraction of *G. lucidum* fruiting body can be used to obtain a triterpenoid-rich anti-tumor agent, which may have potential clinical significance for the treatment of human hepatoma.

## Introduction

*Ganoderma lucidum* (Leys. ex Fr.) Karst. (*G. lucidum*), also known in China as Lingzhi, is a precious traditional Chinese medicine which has been used for more than 2000 years (Li Z. et al., 2020). Its medicinal information was first shown in the book of Shen Nong Ben Cao Jing (Shennong Materia Medica) written in 100 B.C. in China. *G. lucidum* is registered in the Chinese Pharmacopoeia for the efficacy of replenishing qi, tranquilizing the mind, and relieving cough and asthma (Chinese Pharmacopoeia Commission, 2015). In the past few decades, it has been reported that *G. lucidum* has anti-cancer (Li et al., 2013a; Guo et al., 2018; Zhao et al., 2018; Zhu et al., 2018; Acevedo-Díaz et al., 2019), immunomodulatory (Ishimoto et al., 2017), antioxidant (Kan et al., 2015; Bhardwaj et al., 2016), antidiabetic (Pan et al., 2013; Ma et al., 2015), antimicrobial (Gaylan et al., 2018; Naveenkumar et al., 2018), hepatoprotective (Chatterjee and Acharya, 2016), and anti-inflammatory (Cai et al., 2016) effects. Since it has been proved to improve quality of life in cancer patients, *G. lucidum* preparation is widely used in adjuvant therapy for cancer as a traditional Chinese medicine (Bao et al., 2012; Jin et al., 2016). To date, triterpenoid and polysaccharides are considered to be the main anti-tumor components of *G. lucidum* (Ahmad et al., 2020). The ethanol reflux extraction method is commonly used for industrially extraction of triterpenoids from the fruiting body of *G. lucidum*, but it needs longer extraction time and generates organic solvent residue (Zhang et al., 2011). In comparison, supercritical CO_2_ fluid extraction has obvious advantages including short extraction time, low extraction temperature and no solvent residue. It has a good development prospect in the food or pharmaceutical industry (Molino et al., 2018; Jiao et al., 2020). Due to the non-polar nature of CO_2_, supercritical CO_2_ with entrainer was used to extract triterpenoids from the fruiting body of *G. lucidum* at 24 MPa (Lu et al., 2012). Since higher extraction pressure can increase the density of supercritical CO_2_, which enhances its ability to extract liposoluble components, our team has extracted triterpenoid and sterols from *G. lucidum* fruiting body by the technology of high-pressure supercritical CO_2_ extraction without entrainer (Hua et al., 2018). However, the effects of different extraction pressures on the triterpenoid content of *G. lucidum* and the anti-tumor effect of the extracts was not clear. In the present study, we used high-pressure supercritical CO_2_ extraction technology to investigate the effect of supercritical CO_2_ under different extraction pressures on the total triterpenoids content of the extracts. And we further studied the anti-tumor effect of the extracts on human hepatoma cells QGY7703 and SK-Hep1. Our results suggest that within a certain range, the extraction pressure is directly proportional to the triterpenoids content and the anti-tumor effect of supercritical CO_2_ extract of *G. lucidum* fruiting body.

Human hepatoma is a common type of human cancer. It is also a significant cause of cancer-related death throughout the world. Aberrant activation of the Ras/Raf/MEK/ERK pathway has been shown to be involved in the pathogenesis and progression of human hepatoma (Gao et al., 2015). Although it was reported that *G. lucidum* extract inhibited liver cancer growth by arresting cell cycle in G2 phase and inhibiting protein kinase C (Lin et al., 2003). However, to date, the effect of high-pressure supercritical CO_2_ extracts of *G. lucidum* on the Ras/Raf/MEK/ERK signaling pathway in liver cancer cells has not been reported. In the present study, the high-pressure supercritical CO_2_ extracts of *G. lucidum* fruiting body were evaluated for their effects on QGY7703, SK-Hep1 cells and the Ras/Raf/MEK/ERK signaling pathway, which laid a foundation for finding drugs to treat liver cancer.

## Materials and Methods

### Materials and Reagents

The fruiting body of *G. lucidum* was collected from Fujian Xianzhilou Biological Science and Technology Co., Ltd., Fuzhou, China. The fungus was identified as *G. lucidum* (Curtis: Fr.) Karst. (Polyporaceae), and a voucher specimen (no. GL-20140309) has been deposited at the Pharmacognosy Laboratory, School of Pharmacy, Fujian Medical University. The standards of ganoderic acid A, ganodermanontriol and Oleanolic acid are provided by China National Institute of Pharmaceutical and Biological Products.

MTT [3-(4, 5-dimethyl thiazole-2)-2, 5-diphenyltetrazolium bromide] was purchased from Sigma, St Louis, MO, United States. Primary antibodies against Caspase-7, caspase-3, caspase-9, cleaved caspase-9, PARP, cleaved PARP, Ras, C-Raf, p-C-Raf, MEK, ERK, p-MEK, p-ERK and GADPH were obtained from cell signaling Technology, Inc., Danvers, MA, USA. Horseradish peroxidase (HRP) labeled sheep anti-rat/anti-rabbit IgG (secondary antibody), BCA protein quantitative kit, protease inhibitor, phosphatase inhibitor, flow cytometry apoptosis detection kit were all purchased from Roche Diagnostics, Basel, Switzerland. SPF BALB/C nude mice were purchased from Shanghai SLAC Laboratory Animal Co., Ltd., Shanghai, China (certificate No. 2015000560044).

### Preparation and Analysis of Extracts

High-pressure supercritical CO_2_ extraction of *G. lucidum* fruiting body was carried out as described in the literature (Hua et al., 2018). Briefly, the *G. lucidum* fruiting body fine powder 1 kilogram was charged into the supercritical fluid CO_2_ extracting apparatus (Uhde High Pressure Technologies GmbH, Hagen, Germany). The extraction was carried out under different pressures (30, 65, 85, 105 MPa). The extraction conditions also included 50°C of temperature, 4.0 h of extraction time and 12 L/h of CO_2_ flow rate. The extracts were collected from the separation kettle and dried at 65°C. The supercritical CO_2_ extracts obtained at 65, 85, and 105 MPa were named G65, G85, and G105, respectively. However, under 30 MPa extraction pressure of supercritical CO_2_ without ethanol entrainerthe the extract could not be obtained. *G. lucidum* extract (GLE) was obtained from *G. lucidum* fruiting body fine powder 1 kilogram by ethanol through heating reflux in a water bath.

The total triterpenoid contents of *G. lucidum* extracts (G65, G85, G105, and GLE) were determined by Varian Cary 50 ultraviolet-visible spectrophotometry according to the report previously with some modification (Wu et al., 2016). Brieﬂy, the methanol solution of each extract (200 μl, 20 mg/ml) was evaporated in a water bath and mixed with the vanillin-glacial acetic acid (5%, w/v, 0.3 ml) and 1.2 ml of perchloric acid. The mixture was incubated at 70°C for 15 min, cooled for 5 min in an ice water bath and mixed with 8.5 ml of glacial acetic acid. After 15 min, the absorbance was read at 550 nm. Oleanolic acid was used as a reference standard while the total triterpenoid content was expressed as an oleanolic acid equivalent through the calibration curve for oleanolic acid.

The HPLC analysis of *G. lucidum* extracts, ganoderic acid A and ganodermanontriol were determined by the following method. The samples were dissolved in acetonitrile solution and filtered by 0.45 μm membrane. 20 μl supernatant was injected into the HPLC system. Chromatographic conditions: LC-20AD liquid chromatograph (Shimadzu Corporation, Kyoto, Japan) with Kromasil C18 column (4.6 × 250 mm, 5 μm). Mobile phase: acetonitrile (B) - 2% acetic acid water (A), gradient mode : 0 min, A: 65%, B: 35%; 10 min, A: 63%, B: 37%; 30 min, A: 61%, B: 39%; 35 min, A:55%, B: 45%; 50 min, A:38%, B: 62%; 90 min, A:0%, B: 100%. The flow rate was 1.0 ml/min. The column temperature was 30°C. The detection wavelength was 254 nm.

### Cell Culture and Treatment

The two human hepatoma cancer cell lines (QGY7703 and SK-Hep1) were originally obtained from American type culture collection (ATCC). Cells were maintained in DMEM medium containing penicillin (50 U/ml), streptomycin (50 U/ml) and 10% fetal bovine serum (FBS; Germany PAN biotechnology Co., Ltd.) at 37°C in a humidified incubator containing 5% CO_2_.

The cells were treated with 31.25, 62.5, 125, 250, and 500 μg/ml of each *G. lucidum* extract. Each *G. lucidum* extract was dissolved in dimethyl sulfoxide (DMSO). The final concentration of DMSO in each well was less than 0.1% (v/v).

### Cytotoxicity and Colony Formation Assay

The cells were cultured in a 96-well plate at a density of 4×10^3^ cells/well, and then incubated with or without *G. lucidum* extract. The cell viability was determined using an MTT assay to evaluate the cytotoxicity of the extract (Xu et al., 2018).

The colony formation assay was conducted with the previous method (Shi et al., 2018). QGY7703 and SK-Hep1 cells were seeded in 12-well plate at 5 × 10^4^ cells per well. After 12 h of treatment with *G. lucidum* extract, the cells were resuspended. Then, each well was seeded with 500 cells per well in 12-well plate, and cultured in a cell culture box for 14 days to form colony. The cells were washed with PBS, fixed with paraformaldehyde and then stained with 0.5% (v/v) crystal violet. The colony formation was captured via a stereomicroscope (model SZX16) from Olympus, Tokyo, Japan.

### Western Blotting

QGY7703 and SK-Hep1 cells (3 × 10^6^ cells/well in 10 cm^2^ plate) were treated with 150 μg/ml *G lucidum* extract or DMSO for 48 h. Then, total protein was extracted. 20 μg protein was loaded and separated by SDS-PAGE and transferred to polyvinylidene difluoride membranes according to previously procedures (Li et al., 2019). After blocking with 5% non-fat milk at room temperature for 2 h, the membranes were incubated with the indicated primary antibodies overnight at 4°C. The membranes were then washed three times and incubated with peroxidase-conjugated goat anti-rabbit or anti-mouse secondary antibodies, the immunoreactivity was visualized using an ECL Kit (Amersham Pharmacia Biotech, Piscataway, NJ, United States).

### Cell Cycle and Apoptosis Analysis

Cells (3 × 10^5^) were seeded in 6-well plate and treated with *G. lucidum* extracts for 24 h. The cells were harvested, fixed with 3 ml ice-cold 75% ethanol at 4°C for 1 h and stained with 50 µg/ml of propidium iodide (PI). Cell cycle analysis was performed on a flow cytometer from Becton-Dickinson, San Jose, CA as previously described (Yoshioka et al., 2017).

Cells (3 × 10^5^) were seeded in 6-well plate and treated with *G. lucidum* extract for 24 h. Cells were washed with ice-cold PBS, resuspended in 100 µl of binding buffer (1% bovine serum albumin in PBS) and stained with fluorescein isothiocyanate (FITC)-Annexin-V (BD Biosciences, San Jose, CA). The cells were incubated for 15 min at room temperature in the dark. Subsequently, 200 µl of binding buffer containing 20 µg/ml PI was added immediately prior to flow cytometry. The cells were analyzed with FACSCanto cytometer and FACSDiva software (V.5.0.2) from BD Biosciences, San Jose, CA. Early and late apoptosis or necrosis were evaluated (Lu et al., 2019).

### Anti-Tumor Experiment of Extracts in Xenografts Model

G85 was dissolved in a mixture of ethanol and 0.5% carboxymethyl cellulose saline (1:9, V/V). The dosage of G85 were 300 and 75 mg/kg by gavaged on the basis of preliminary experiments.

Athymic nude mice (BALB/c-nu, 6–8 weeks of age; male, body weight: 20.0 ± 2.0 g) were obtained from Shanghai SLAC laboratory animal Co., Ltd. (Shanghai, China) and randomly divided into six groups: negative control (gavage daily with vehicle), positive control (intraperitoneal injection 30.0 mg/kg cyclophosphamide: every 2 days) and G85 treatment (gavage daily with 75 and 300 mg/kg). Mice in each group were inoculated with about 20 mm^3^ volume of SK-Hep1 solid tumors on the right forearm. When the tumor grew to 100 mm^3^, then the mice were administrated for 43 days, their body weight and tumor volume was measured every three days. At the end of experiment, tumors were excised and weighed (Huang et al., 2014). All animal experiments were approved by the Laboratory Animal Welfare & Ethics Committee, Fujian Medical University (grant number FJMU IACUC 2017-0093).

### Statistical Analysis

All data were presented as the mean ± SD. Student’s t-test was used to valuate the mean difference between the two groups. *p* < 0.05 was considered significant.

## Results

### Chemical Analysis of the Extracts

**FIGURE 1:**
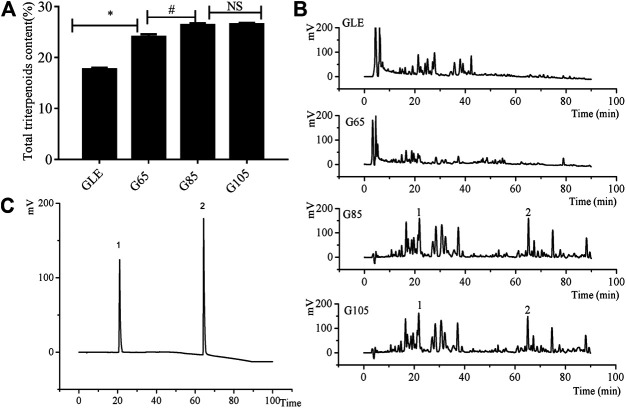
The chemical analysis of the extracts. **(A)** The total triterpenes content of *G* *. lucidum* extracts **(B)** HPLC chromatograms of *G* *. lucidum* extracts **(C)** HPLC chromatograms of reference substance. 1) ganoderic acid A, 2) ganodermanontriol. Data were shown as mean ± SD (*n* = 3), *p < 0.05 vs GLE, ^#^ *p* < 0.05 vs G65, NS means no significant difference.

The extraction yield of G65, G85, G105, and GLE was 3.82%, 4.81%, 5.66%, and 5.28% respectively. Though the extraction yield of G65 and G85 was lower than that of GLE, the total triterpenoid content of G65 and G85 was higher than that of GLE. It was worth noting that the total triterpenoid content of G85 was 1.34 times that of GLE. Althought the extraction yield of G105 was highest among the all extracts, there was little difference in the total triterpenoids content between G85 and G105, which suggested that the increase of extraction pressure could improve the extraction yield, however, when the pressure was over 85 MPa, the content of total triterpenoids tends to reach saturated state (Figure 1A). When the extraction pressure was too high, the solubility of impurities could increase, while the solubility of the target extract did not change much, which led to a decrease in the proportion of active substances in the extract.

HPLC chromatogram of the extracts (Figure 1B) showed that the retention time of chemical components in the extracts was mainly distributed in the two periods: 0–40 and 60–90 min. Compared with G65 and GLE, the number and area of chromatographic peaks of G85 within a retention time of 60–90 min was significantly increased. However, there was no significant difference between G85 and G105 in the number and area of chromatographic peaks. As representative triterpenoids in the extracts of *G. lucidum*, ganoderic acid A and ganodermanontriol were determined with HPLC (Figures 1B,C). The results showed that all extracts especially in G85 and G105 contained ganoderic acid A (retention time 21.05 min) and ganodermanontriol (retention time 64.33 min). From the chromatogram, it was obvious that the content of ganoderic acid A and ganodermanontriol of G85 was higher than that of GLE and G65 but was basically the same as that of G105.

### Antiproliferative Effect of the Extracts on Hepatoma Carcinoma Cells Enhanced With the Increase of Extraction Pressure

**FIGURE 2:**
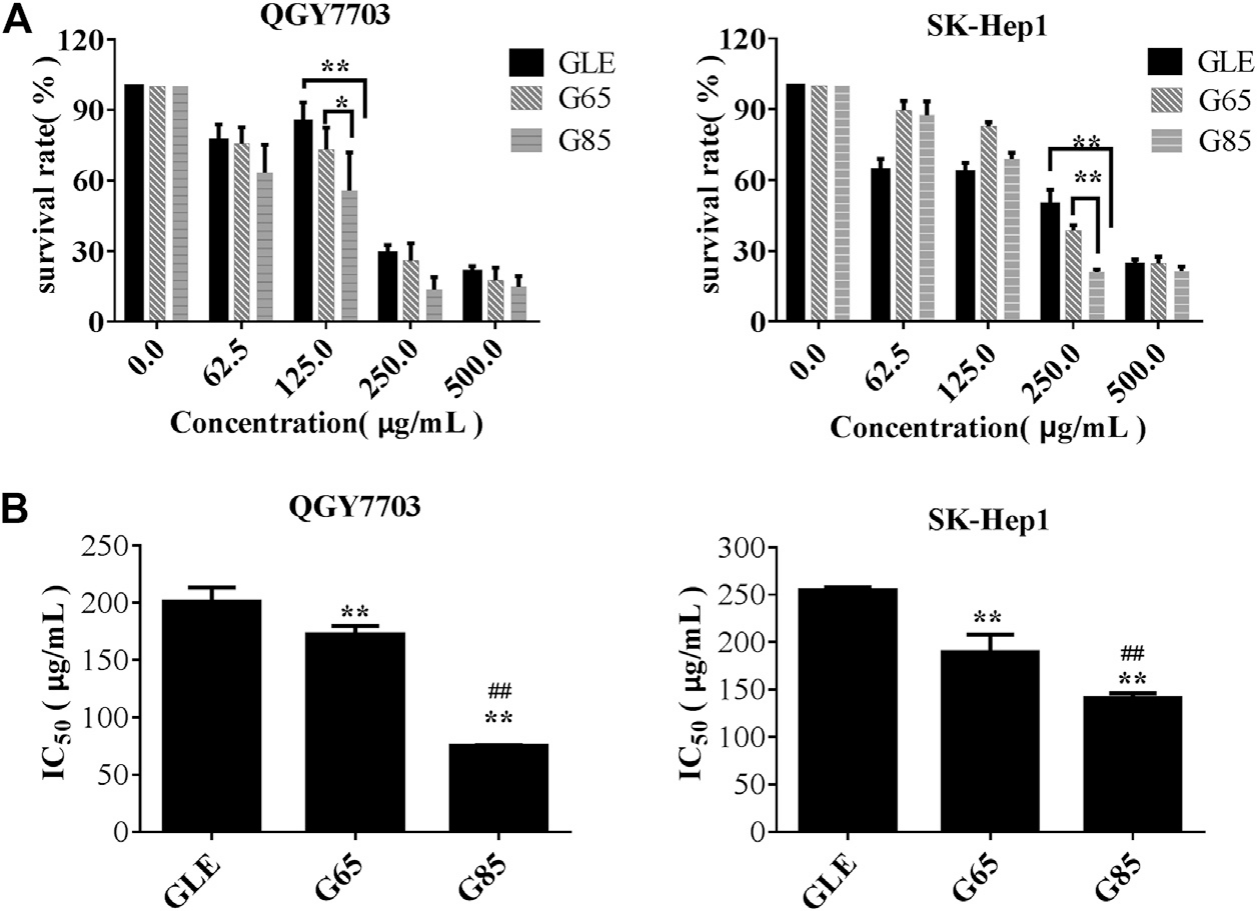
The cytotoxicity of *G* *. lucidum* extracts on human hepatoma carcinoma. **(A)** QGY7703, SK-Hep1 cells were treated with GLE, G65, and G85 for 72 h and cell viability measured by MTT and expressed as a percentage of vehicle control. **(B)** The IC_50_ value of *G* *. lucidum* extracts on QGY7703, SK-Hep1 cells after exposure of 72 h (*n* = 3). Data were shown as mean ± SD (*n* = 3), **p < 0.01 vs GLE, ^##^ *p* < 0.01 vs G65.

**FIGURE 3:**
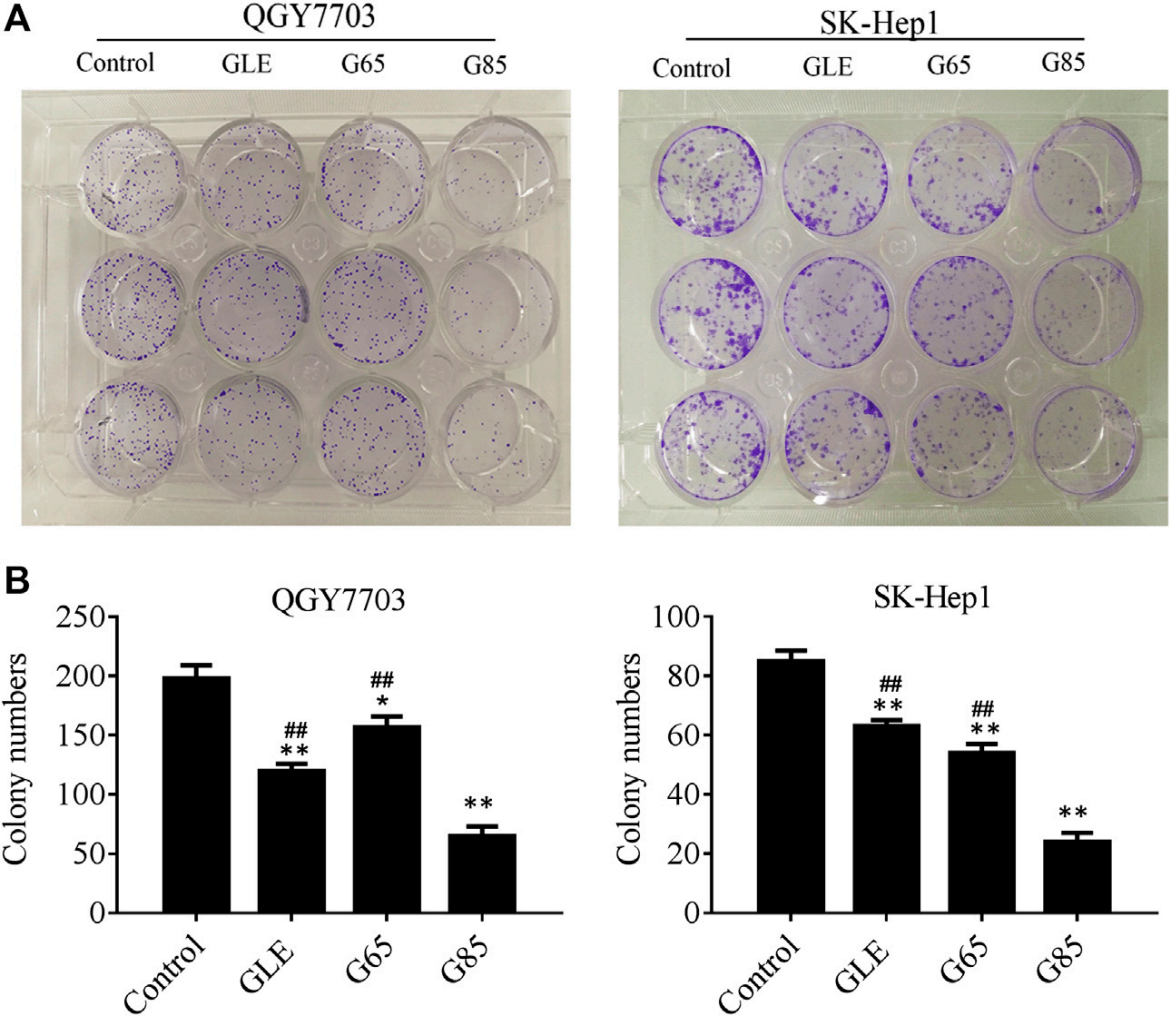
The antiproliferative activity of *G. lucidum* extracts on human hepatoma carcinoma *in vitro*. **(A)** QGY7703 and SK-Hep1 cells were treated with GLE, G65, and G85 for 12 h, and cultured 14 days to form colony. **(B)** The colony number of QGY7703 and SK-Hep1 cells treated with GLE, G65, and G85 for 14 days. Data were shown as mean ± SD (*n* = 3), *p < 0.05, **p < 0.01, vs control. ^#^ *p* < 0.05, ^##^ *p* < 0.01, vs G85.

We found that the total triterpene content increased with the increase of extraction pressure, so does the anti-tumor effect of the extract also enhance? Therefore, we compared the anti-tumor effect *in vitro* among different high-pressure extracts and ethanol reflux extract. Cell viability was measured after treatment with different concentrations of GLE, G65, G85, and G105 for 72 h in the human hepatoma cells (QGY7703 and SK-Hep1). The viability of QGY7703 and SK-Hep1 cells treated with G85 was found to be significantly lower than that treated with G65 and GLE, respectively (Figure 2A), whereas the viability of QGY7703 and SK-Hep1 cells treated with G105 was not clearly different from that treated with G85 (data not shown). The IC_50_ of GLE, G65 and G85 on QGY7703 cells were 200.90 ± 12.58, 172.32 ± 7.59, and 75.34 ± 0.63 μg/ml respectively, while those on SK-Hep1 cells were 255.05 ± 3.07, 189.45 ± 18.67, and 141.12 ± 5.09 μg/ml, respectively (Figure 2B). These data showed that the order of cytotoxicity of extracts on hepatoma carcinoma cells was G85 > G65 > GLE, and colony formation of the extracts on QGY7703 and SK-Hep1 cells also revealed the similar antiproliferative effects (Figures 3A,B). The results indicated that the antiproliferative potency of the high-pressure supercritical CO_2_ extracts on human hepatoma carcinoma cells *in vitro* was consistent with their triterpenoid content, suggesting that with the increase of extraction pressure, the triterpenoid content of the extracts increased, and the antiproliferative activity of the extracts also enhanced.

### Extracts Arrested Cell Cycle in G2/M and Induced Apoptosis on Human Hepatoma Carcinoma Cells

**FIGURE 4:**
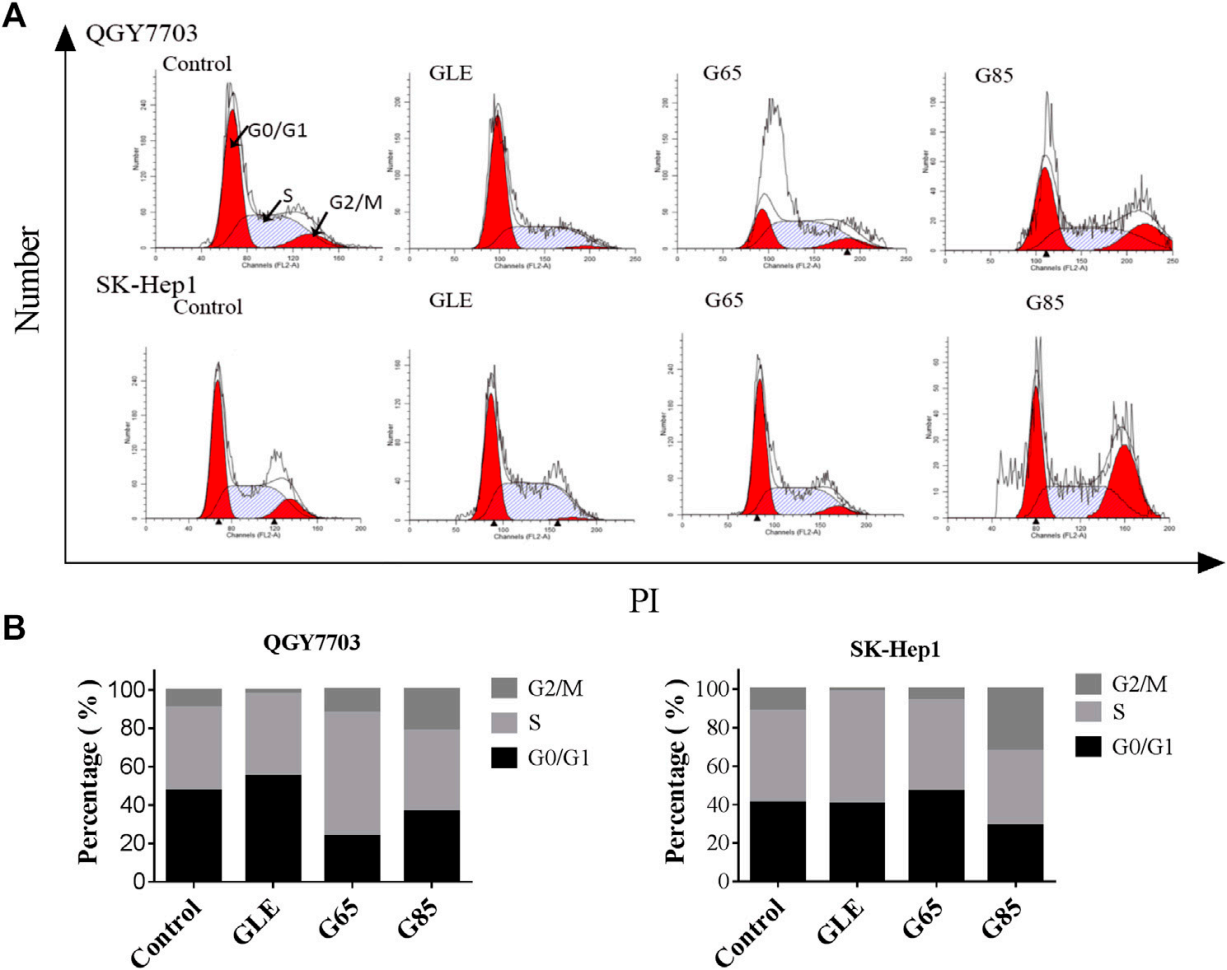
*G* *. lucidum* extracts arrested cell cycle in G2/M. **(A)** QGY7703 and SK-Hep1 cells treated with GLE, G65, and G85 (150 μg/ml) for 24 h, ethanol fixation and PI single staining were performed, and cell cycle was detected by flow cytometry. **(B)** Percentage of QGY7703 and SK-Hep1 cells in different cell cycle phases was shown.

Compared with vehicle control, QGY7703 and SK-Hep1 cells treated with GLE, G65, and G85 (150 μg/ml) for 24 h displayed obvious cell cycle arrest in the G2/M phase. The increase of cell population in the G2/M phase was accompanied by a concomitant decrease of cell population in the S and G0/G1 phases. However, among these three extracts, G85 had the most significant effect on cell cycle arrest, particularly for SK-Hep1 cells (Figure 4).

**FIGURE 5:**
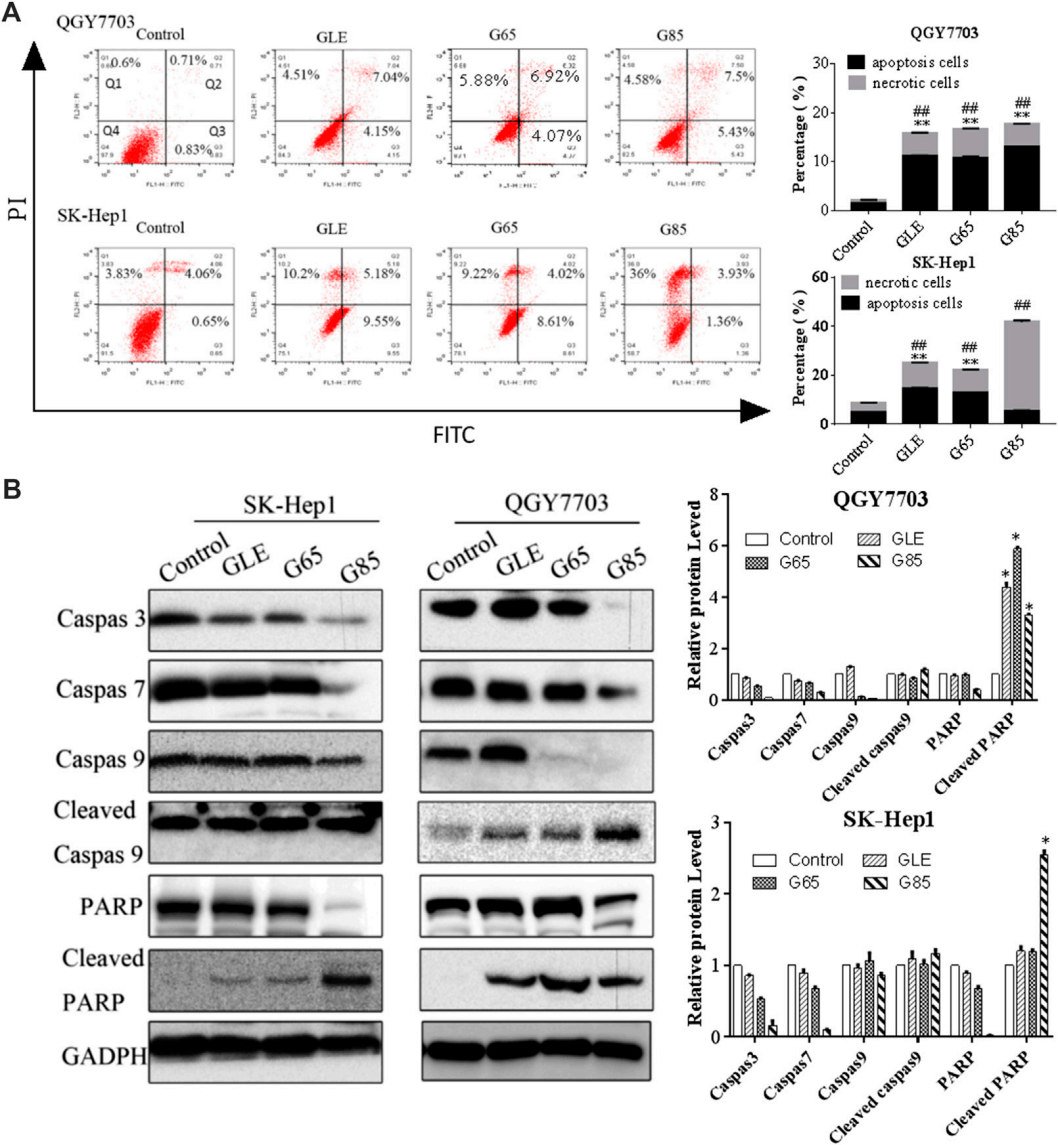
Effect of *G* *. lucidum* extracts on QGY7703 and SK-Hep1 cells apoptosis. **(A)** QGY7703 and SK-Hep1 cells were treated with GLE, G65 and G85 (150 μg/ml) for 24 h and analyzed for apoptotic and necrotic cell death using an Annexin-V: FITC Apoptosis Detection Kit. **(B)** QGY7703 and SK-Hep1 cells were treated with GLE, G65, G85 (150 μg/ml) for 48 h. The expression of Caspase 9, 3, and 7, PARP, cleaved Caspase 9 and cleaved PARP were determined by western blot. Data were shown as mean ± SD. *p < 0.05 (cleaved PARP),**p < 0.01 (apoptosis rate), ^##^ *p* < 0.01 (necrosis rate), vs control.

QGY7703 and SK-Hep1 cells were treated with GLE, G65, and G85 (150 μg/ml) for 24 h and analyzed for apoptotic cell death using an Annexin-V: FITC Apoptosis Detection Kit. The results showed that GLE, G65, and G85 all induced apoptosis and necrosis in SK-Hep1 cells and QGY7703 cells, among which G85 showed the strongest cytotoxicity. The apoptosis induction of G85 on QGY7703 cells was significantly stronger than that of SK-Hep1 cells, while the necrosis effect of G85 on SK-Hep1 cells was more obvious. The above results suggested that cell necrosis and apoptosis caused by the extract accounted for their decrease of cells viability (Figure 5A). Western blot analysis revealed that G85 could significantly down-regulate caspase 3 and 7 in both cell lines, and decrease caspase 9, increase the cleaved caspase 9 in QGY7703 cells, which was consistent with its more significant apoptosis induction in QGY7703 cells (Figure 5B). It has been reported that excessive activation of PARP can cause tumor cell necrosis (Sosna et al., 2014). G85 significantly downregulated PARP and upregulated cleaved PARP, which suggested that G85 could induce necrosis on hepatocellular carcinoma cells, especially on SK-Hep1 cells.

### Extracts Significantly Inhibited the Ras/Raf/MEK/ERK Signaling Pathway

**FIGURE 6:**
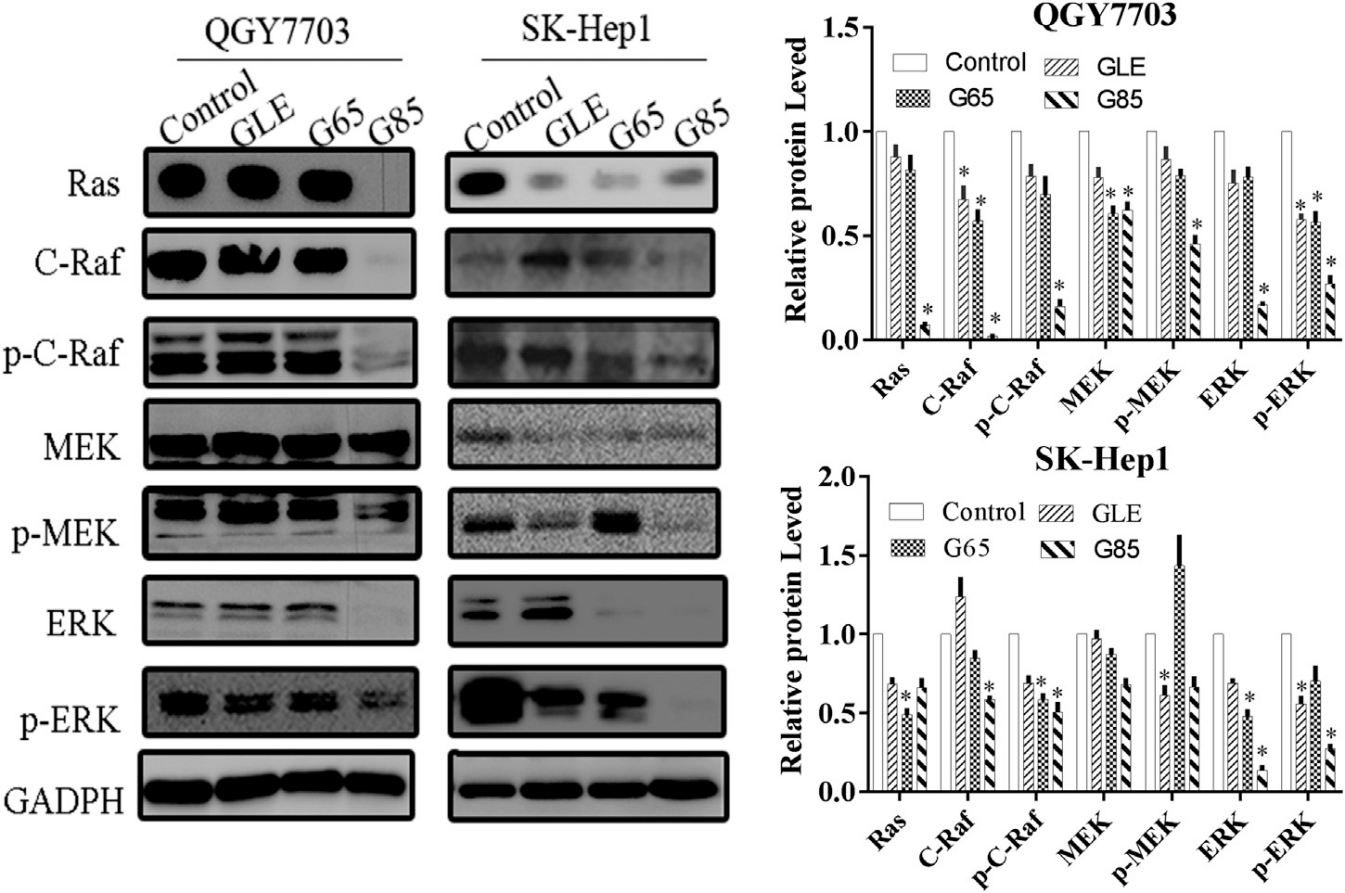
*G. lucidum* extracts significantly inhibited Ras/Raf/MEK/ERK signaling pathway. QGY7703 and SK-Hep1 cells were treated with GLE, G65, and G85 (150 μg/ml) for 48 h. The expressions of Ras, c-Raf, p-c-Raf, MEK, *p*-MEK, ERK, and *p*-ERK were determined by western blotting. The western blot density was analyzed, and the values of each protein were normalized with the values of the internal reference protein GADPH, and further compared with the control group. Data were shown as mean ± SD. *p < 0.05, vs control.

The Ras/Raf/MEK/ERK signaling pathway is involved in multiple activities in the cell cycle and is usually in an activated status in most advanced hepatocellular carcinoma cases (Gao et al., 2015), and plays critical roles in prevent apoptosis (McCubrey et al., 2006). The inhibition of this signaling pathway may effectively suppress the growth of hepatocellular carcinoma cells, which has been evidenced by many recent studies (Zhang et al., 2016; Zhou et al., 2017). Therefore, we assessed the effect of the extracts on Ras/Raf/MEK/ERK signaling pathways by Western blot. As expected, after QGY7703 and SK-Hep1 cells exposed to the 150 μg/mL extracts for 48h, protein levels of Ras/Raf/MEK/ERK observably decreased, while the phosphorylation of Raf, MEK and ERK were inhibited by G85 to a greater extent than by GLE and G65 (Figure 6), which was consistent with their inhibition of proliferation and induction of apoptosis of human liver cancer cells. These data implied that the cytotoxicity of extracts on hepatocellular carcinoma cells may be associated with inhibition of the Ras/Raf/MEK/ERK signaling pathway.

This data suggested that high-pressure supercritical CO_2_ extracts of *G. lucidum* fruiting body, especially G85, may have potential clinical value in the treatment of human hepatoma.

### G85 Significantly Inhibited Tumor Growth in SK-Hep1 Cell Xenograft Model

Given G85 had the highest content of *G. lucidum* triterpenoid and the strongest anti-tumor activity *in vitro* among high-pressure supercritical CO_2_ extracts of *G. lucidum* fruiting body, we selected G85 to test its anti-tumor effect on SK-Hep1 cells *in vivo*. In the previous study, we found that the triterpenoid extract of *G. lucidum* had a protective effect on acute liver injury induced by carbon tetrachloride and alcohol in mice at a dose of 1 g/kg (Li et al., 2013b), indicating that the triterpenoid extract of *G. lucidum* has liver protective effect in this dosage. In this study, we chose 75 and 300 mg/kg doses of G85 based on the preliminary experiment to observe its anti-tumor effect *in vivo*.

**FIGURE 7:**
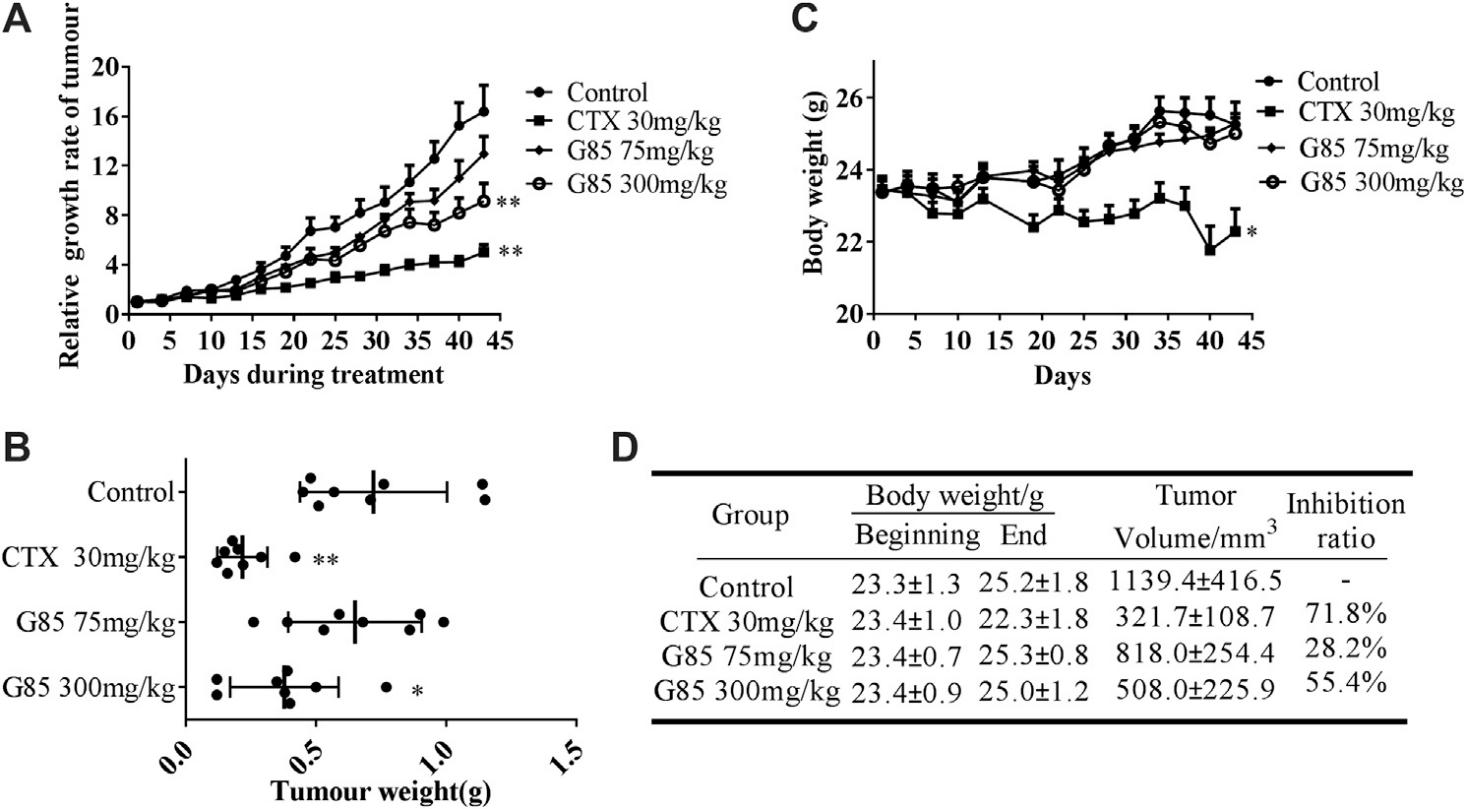
The antitumor effect of G85 in the SK-Hep1 cell xenograft model *in vivo*. **(A)** The tumor growth curve, drawn according to the average percentage change of the tumor volume **(B)** The tumor weight of SK-Hep1 xenograft at the end of treatment in each group (*n* = 8). **(C)** The curve of mouse body weight measured every 3 days **(D)** Data of body weight before and after course of treatment and tumor inhibition at the end of treatment. Data were shown as mean ± SD.*p < 0.05, **p < 0.01, vs control.

SK-Hep1 human liver cancer xenograft model was established to assess the anti-tumor effect of G85 *in vivo* at two doses (75 and 300 mg/kg i.g., qd). CTX (30 mg/kg i.p., q2d) was used as a positive control. The results demonstrated that G85 inhibited tumor growth in the SK-Hep1 xenograft model in a dose-dependent manner (Figures 7A,B). Compared with the vehicle group, 75 and 300 mg/kg doses of G85 showed tumor inhibition by 28.2% and 55.4%, respectively. Compared with the control group, the body weight of mice in each dose group of G85 showed no significant change, while that in CTX group was significantly lower than the control, indicating that CTX has certain toxicity on mice, while G85 in this dose range has no obvious toxicity on mice (Figures 7C,D).

## Discussion

There are many methods to extract triterpenoids from *G. lucidum*, such as solvent extraction methods (Ruan et al., 2014), ultrasonic extraction methods (Oludemi et al., 2018) and microwave extraction methods (Fu et al., 2010). Some researchers also used supercritical CO_2_ extraction technology to extract triterpenoids from *G. lucidum* spore powder (Li et al., 2016). The principle of supercritical CO_2_ extraction technology is that when CO_2_ is in a critical state, it has the characteristics of weak intermolecular force and high density, which makes CO_2_ in the critical state have high mass transfer rate, strong permeability, and no interphase effect. These advantageous characteristics help to improve the solubility of the medium and save energy. At the same time, the critical CO_2_ has unusually high compressibility. Near the critical point, slight changes in pressure or temperature cause obvious changes in density of CO_2_, so that the dissolution of CO_2_ can be changed simply by adjusting the temperature or pressure of the system to improve the selectivity of extraction (Khaw et al., 2019). Therefore, increasing the solubility of critical CO_2_ by increasing the extraction pressure can become the research direction of process optimization. However, the effect of supercritical CO_2_ extraction pressures on the content of triterpenoid and anti-tumor activity in *G. lucidum* extract remains to be explored. In this study, we analyzed the differences in composition of *G. lucidum* extract obtained with supercritical CO_2_ at different pressures. The *G. lucidum* extract could be collected only When the extraction pressure was up to 65 MPa. With the extraction pressure increasing to 85 MPa, the total triterpenoid content of G85 was significantly higher than that of G65. However, as the pressureat up to 105 MPa, the total triterpenoid content of the extract was only slightly higher than that of G85. Therefore, the optimal pressure of supercritical CO_2_ extraction for triterpenoids has a certain range. Appropriate high pressure such as 85 MPa in this work for supercritical CO_2_ extraction is beneficial to the enrichment of triterpenoids in supercritical extract. It is well known that triterpenoids are one of the main anti-tumor active components of *G. lucidum* (Li, et al., 2019). The triterpenoid content of G85 is relatively high in our extracts, which may account for be the its the most strong antitumor activity in the extracts.

Some researchers had divided the *G. lucidum* extract into neutral and acidic components, and found that the neutral component had strong tumor cytotoxicity (Lu et al., 2012; Li P. et al., 2020). We also separated the neutral component(G85NC) and the acid component(G85AC) from G85. The cytotoxicity assay showed that G85NC had more strong activity than G85AC in QGY7703 cells, and its IC_50_ value was only 0.15 times that of G85AC (Supplementary Figure S1). Taken together, we found that the anti-tumor active fraction of G85 mainly existed in the neutral component (G85NC), which was consistent with the results of previous studies (Gao et al., 2006; Chen et al., 2017). However, which compounds in neutral component of G85 play a major role for the anti-tumor effect is worth further study.

*G. lucidum* extract has been shown to have anti-tumor effect in various cell lines and animal models (Qu et al., 2014; Wang et al., 2016). There are several events involving in the anti-tumor effect of *G. lucidum* extracts such as blocking cell cycle (Zolj et al., 2018), inducing apoptosis and autophagy (Cheng and Xie, 2019), inhibiting tumor metastasis and angiogenesis (Li et al., 2012). The Ras/Raf/MEK/ERK signaling pathway is one of the vital pathways regulating cell proliferation and apoptosis in tumor cells, therefore its inhibition may be accout for suppression of cell growth and sensitivity to apoptosis for tumor cells (McCubrey et al., 2006; Wen et al., 2019). Ganoderan, one of the components of *G. lucidum* polysaccharides, was reported to regulate the growth, motility and apoptosis of non-small cell lung cancer cells through the Ras/Raf/MEK/ERK signaling pathway (Wang et al., 2019). In our study, we first found that G85, a triterpenoid-rich extract with high-pressure supercritical CO_2_ from *G. lucidum*, could significantly inhibit the proliferation and induce apoptosis of liver cancer cells via suppression of Ras/Raf/MEK/ERK signaling pathway. G85 may have potential clinical value in the treatment of human hepatoma and be worth further study.

## Conclusion

Within a certain range, the extraction pressure is directly proportional to the triterpenoids content and anti-tumor effect of *Ganoderma lucidum* supercritical CO_2_ extract. The *Ganoderma lucidum* extract G85 obtained with high-pressure supercritical extraction at 85 MPa has high triterpenoid content and strong anti-tumor effect. The anti-tumor effect of G85 was associated with its inhibition of Ras/Raf/MEK/ERK signaling pathway in liver cancer cells. Therefore, the high-pressure supercritical CO_2_ extraction of *Ganoderma lucidum* fruiting body can be used to obtain a triterpenoid-rich anti-tumor agent, which may have potential clinical value in the treatment of human hepatoma.

## Data Availability Statement

The original contributions presented in the study are included in the article/Supplementary Material, further inquiries can be directed to the corresponding authors.

## Ethics Statement

The animal study was reviewed and approved by the Laboratory Animal Welfare and Ethics Committee, Fujian Medical University.

## Author Contributions

JX provided funding and design for the work. ZZ and YL coordinated technical support. LZ and PL wrote the manuscript. LZ, MW, and PL performed the experiments. JZ collected the samples. YZ and WY analyzed and processed the data. All authors read and approved the final manuscript.

## Funding

This study was supported by grants from National Natural Science Foundation of China (Grant Nos. 81973364 and 81202560), the Chinese medicine modernization project of Ministry of science and technology of China (Grant No. 2019YFC1710503) and the Natural Science Foundation of Fujian Province of China (Grant Nos. 2020J01624 and 2020J01617).

## Conflict of Interest

Author LZ, YZ, YL and WY were employed by company Fujian Xianzhilou Biological and Technology Co., Ltd., Fuzhou, China.

The remaining authors declared that the research was conducted in the absence of any commercial or financial relationships that could be construed as a potential conflict of interest.
