# Supplementary figures and images for: High-Pressure Supercritical CO2 Extracts of Ganoderma lucidum Fruiting Body and Their Anti-hepatoma Effect Associated With the Ras/Raf/MEK/ERK Signaling Pathway
Source: Front Pharmacol. 2020 Dec 14;11:602702. doi: 10.3389/fphar.2020.602702 (PMC7768272; doi:10.3389/fphar.2020.602702)

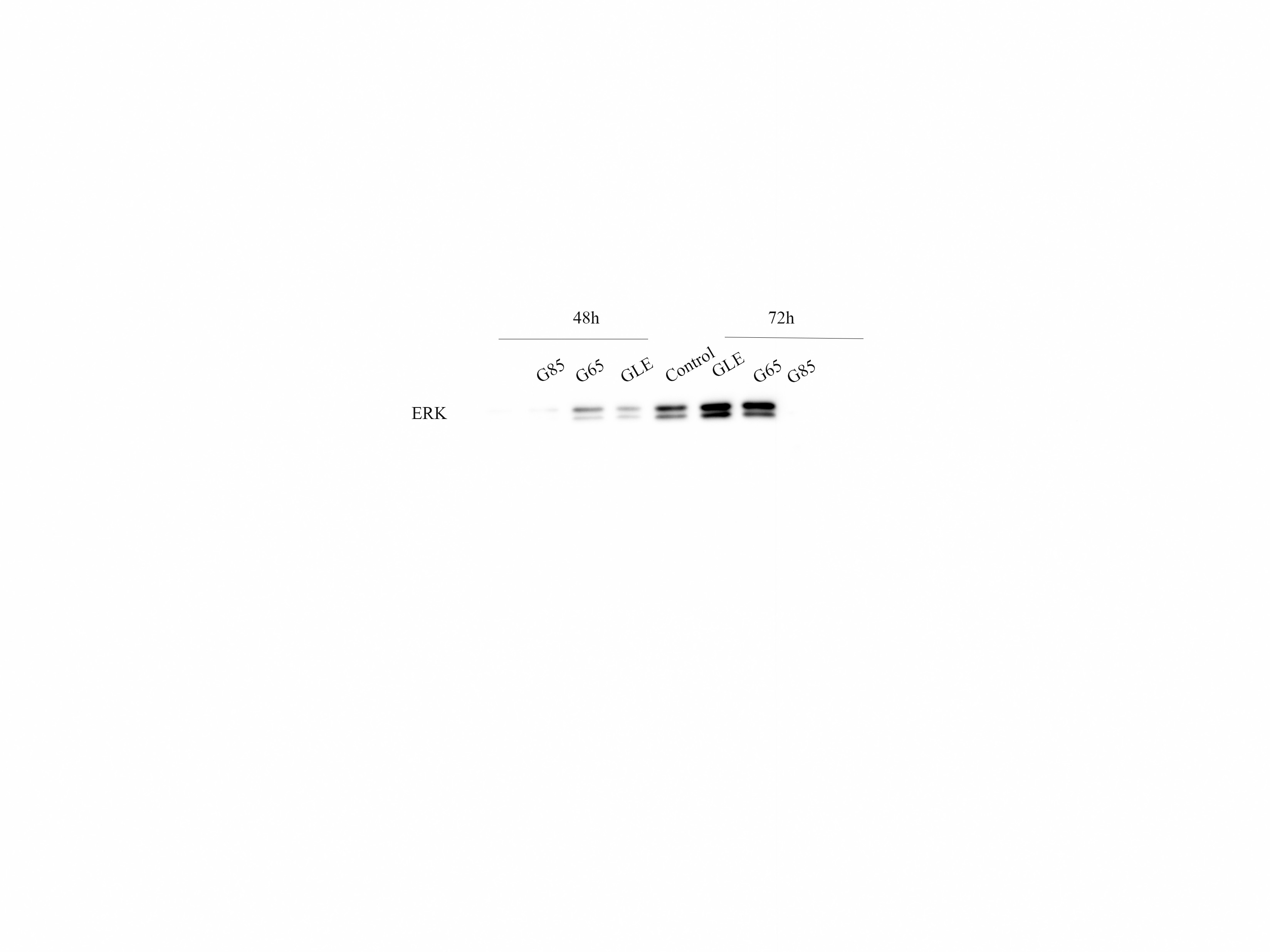

Supplement: Supplementary file 1 [file datasheet1.zip › SK-Hep1 ERK.jpg]

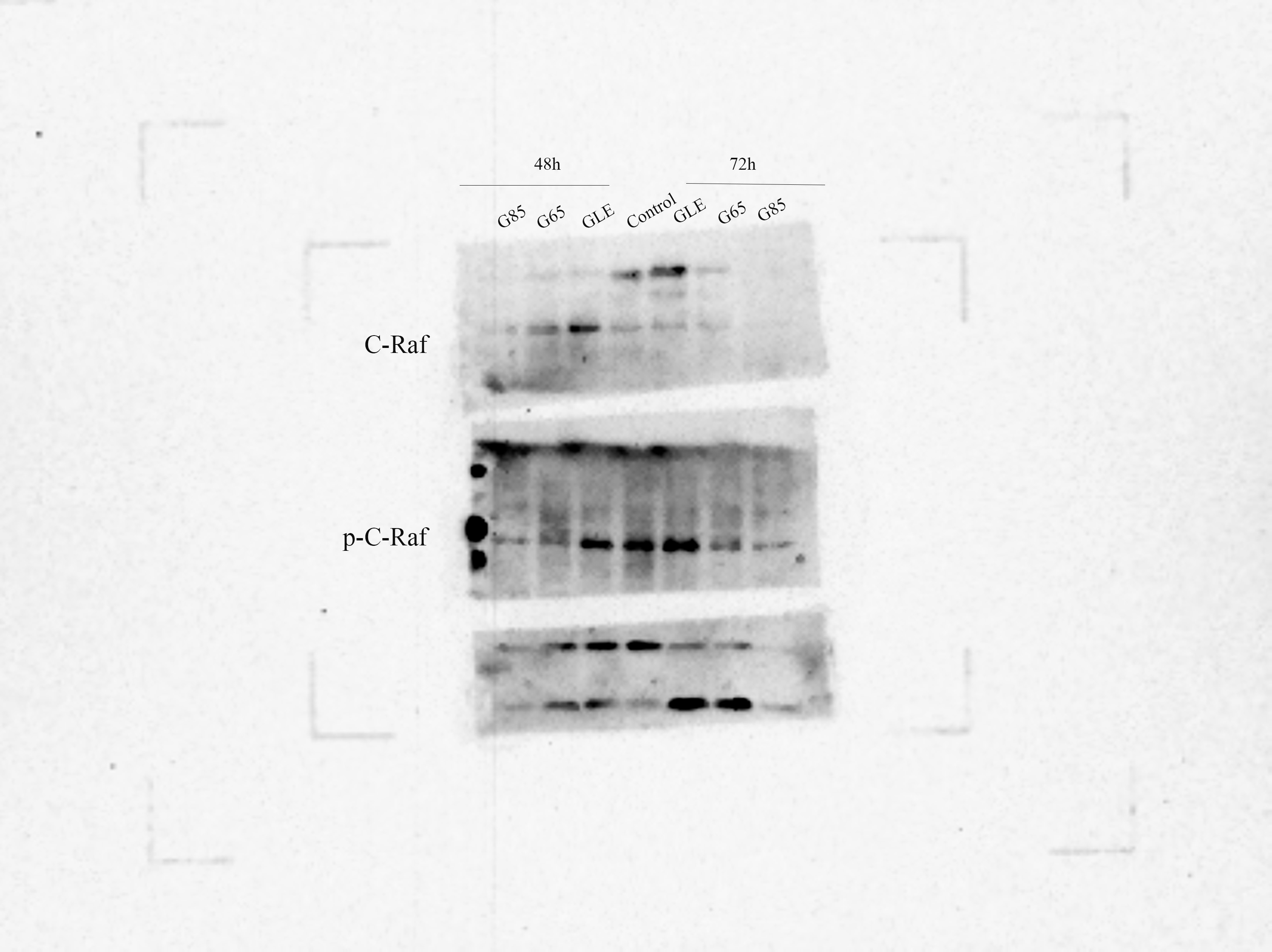

Supplement: Supplementary file 1 [file datasheet1.zip › SK-Hep1 C-Raf, p-C-Raf, MEK(not used).jpg]

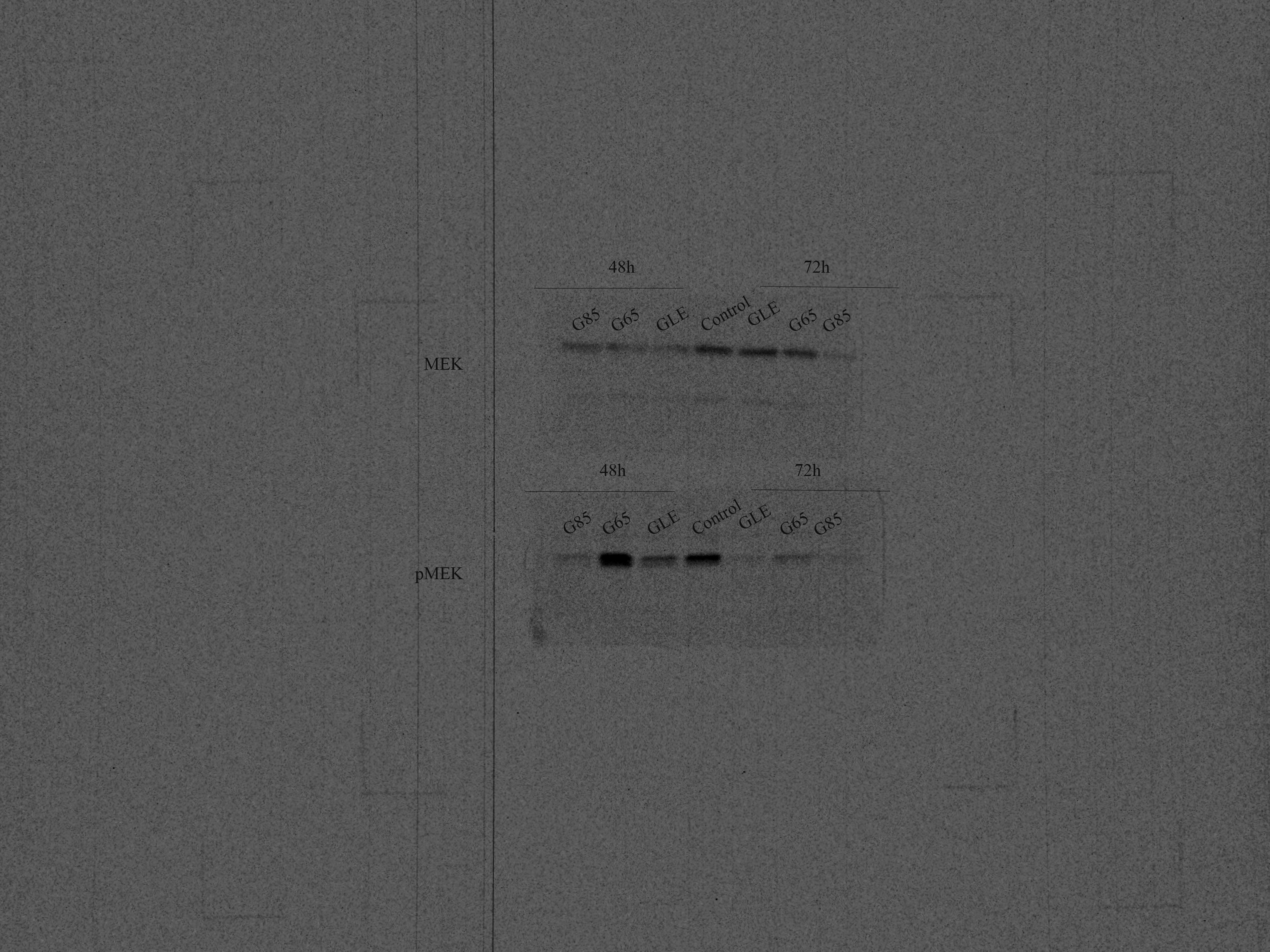

Supplement: Supplementary file 1 [file datasheet1.zip › SK-Hep1 MEK, p-MEK.jpg]

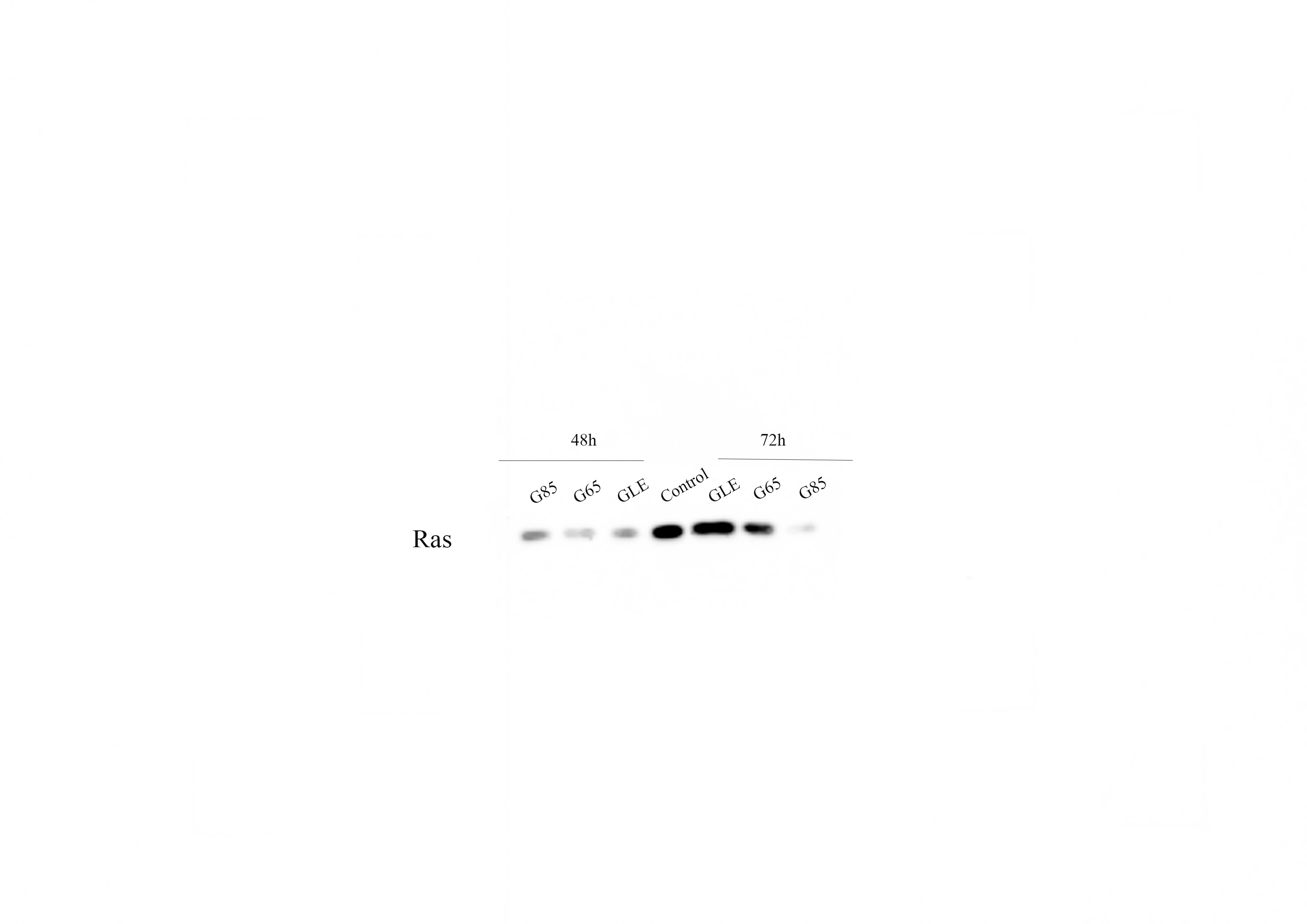

Supplement: Supplementary file 1 [file datasheet1.zip › SK-Hep1 Ras.jpg]

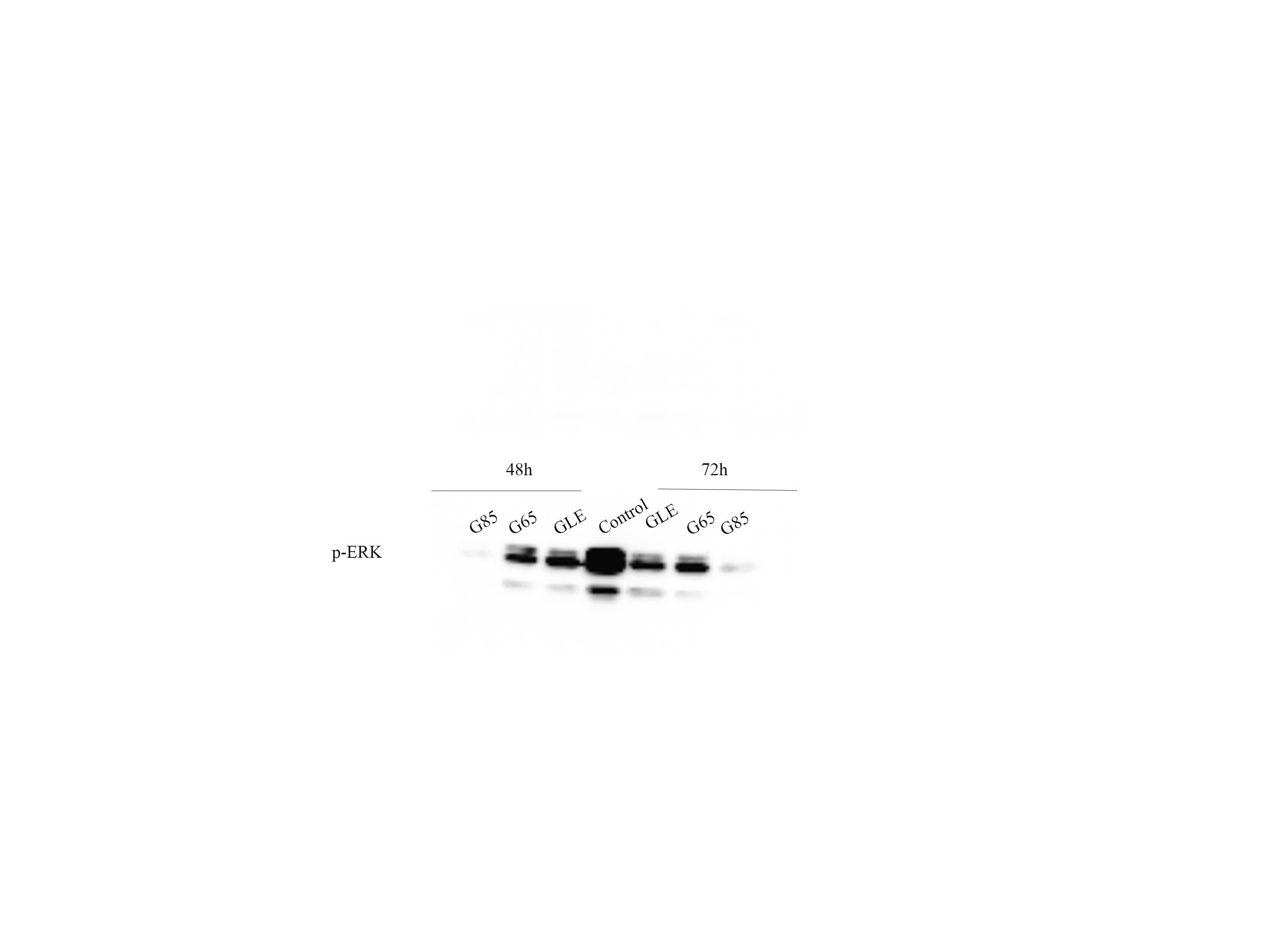

Supplement: Supplementary file 1 [file datasheet1.zip › SK-Hep1 p-ERK.jpg]

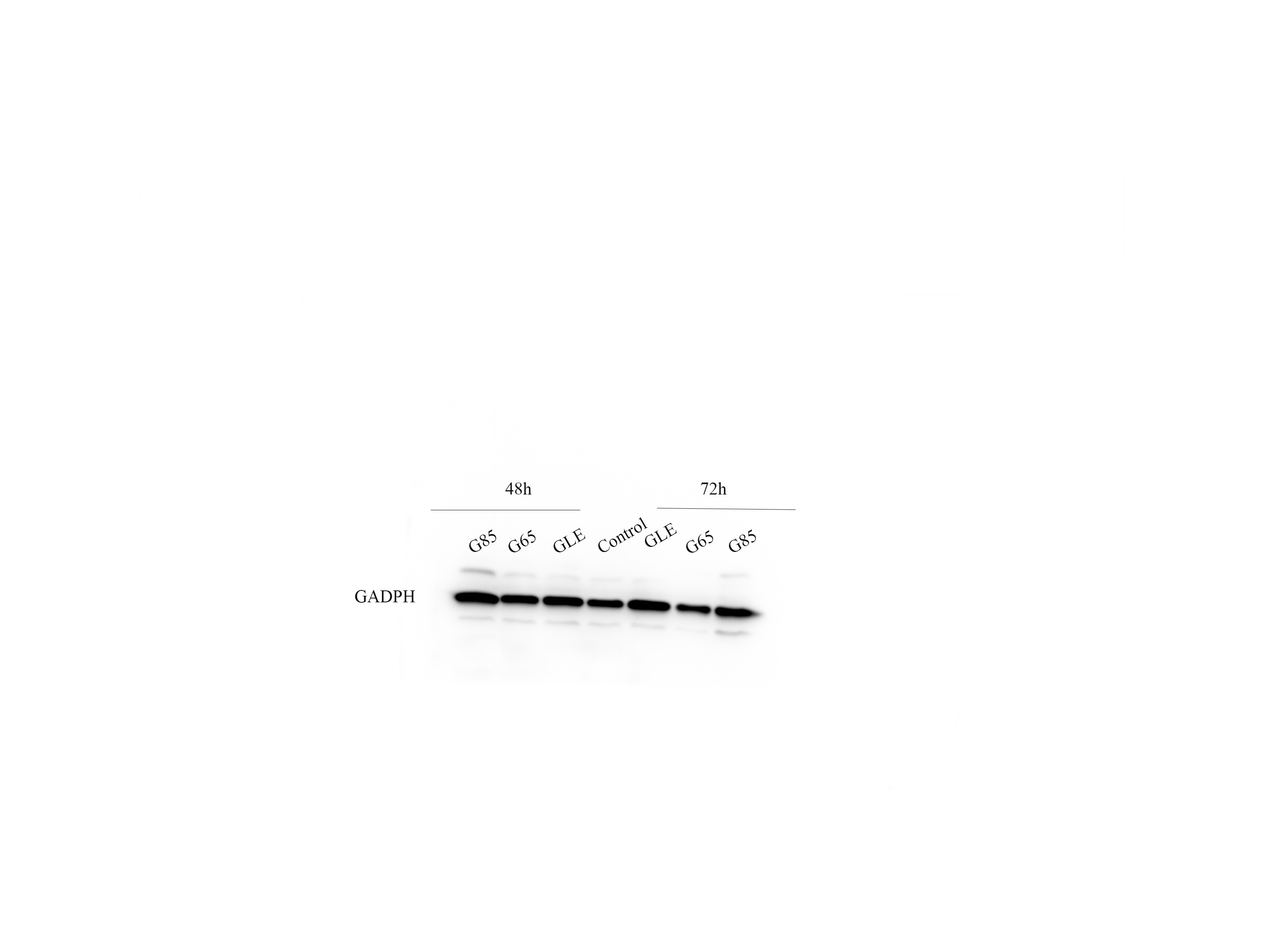

Supplement: Supplementary file 1 [file datasheet1.zip › SK-Hep1 GADPH (MAPK Signaling pathways).jpg]

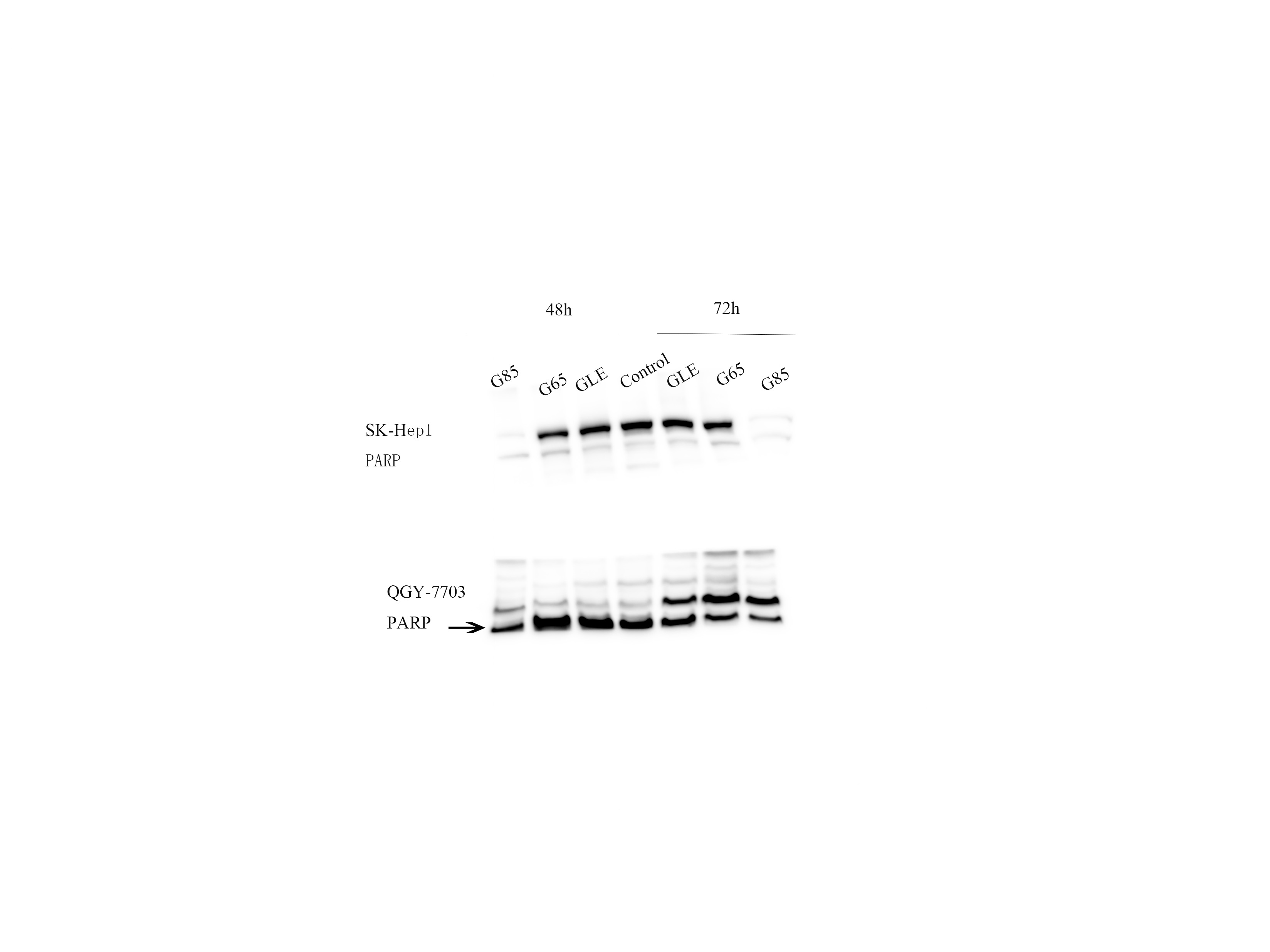

Supplement: Supplementary file 2 [file datasheet2.zip › SK-Hep1 PARP.jpg]

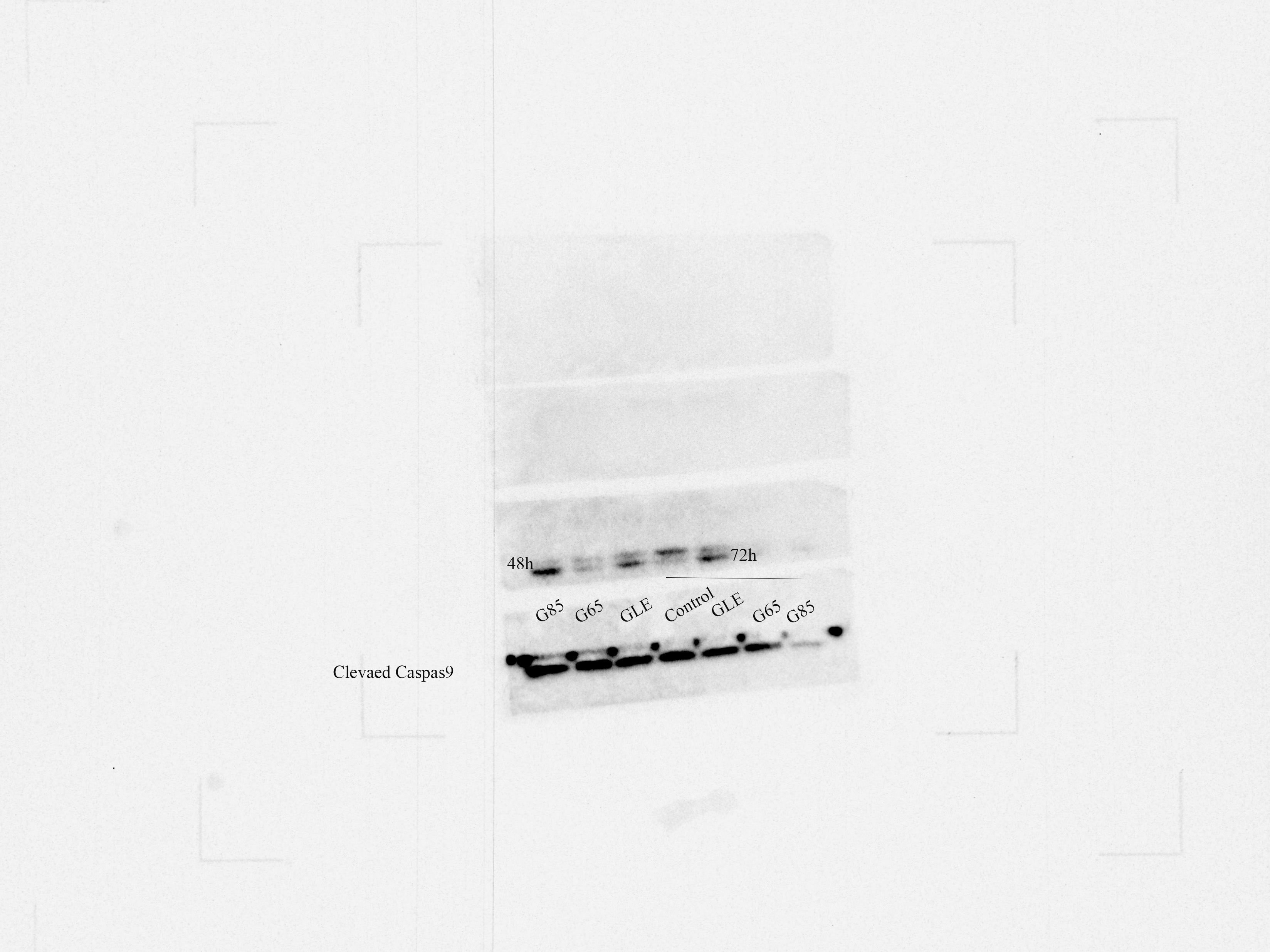

Supplement: Supplementary file 2 [file datasheet2.zip › SK-Hep1 Cleaved Caspas9.jpg]

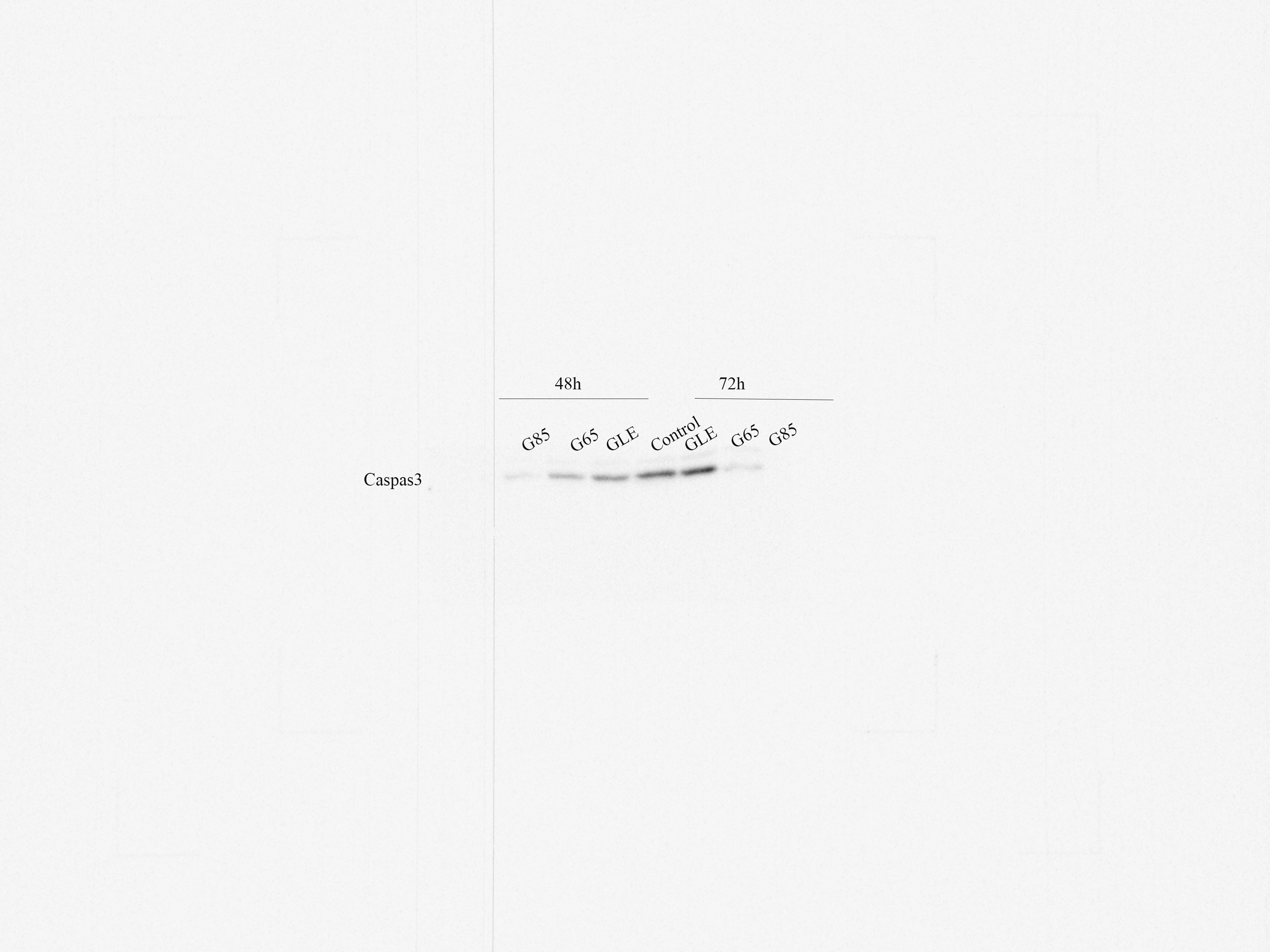

Supplement: Supplementary file 2 [file datasheet2.zip › SK-Hep1 Caspas3.jpg]

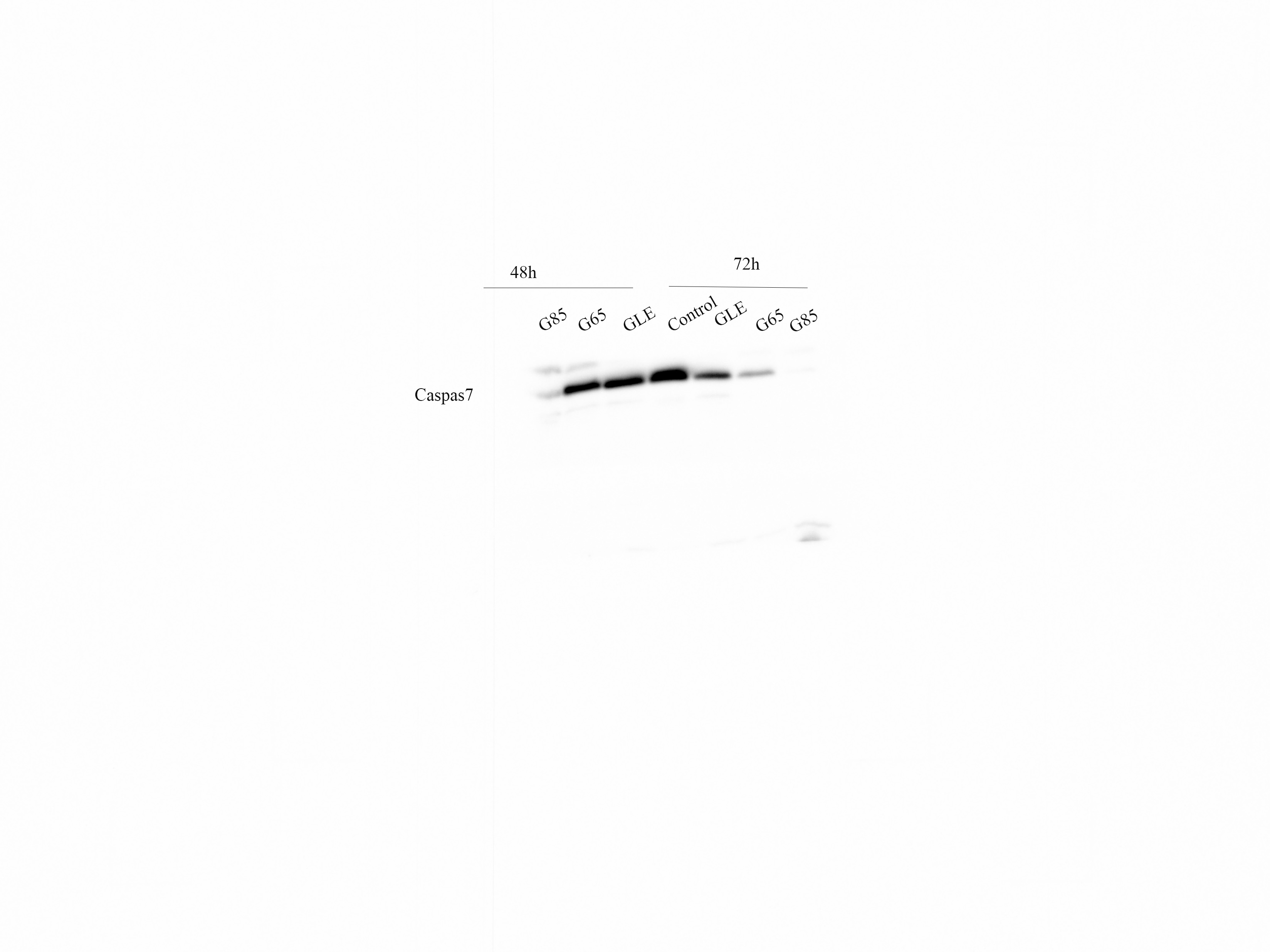

Supplement: Supplementary file 2 [file datasheet2.zip › SK-Hep1 Caspas7.jpg]

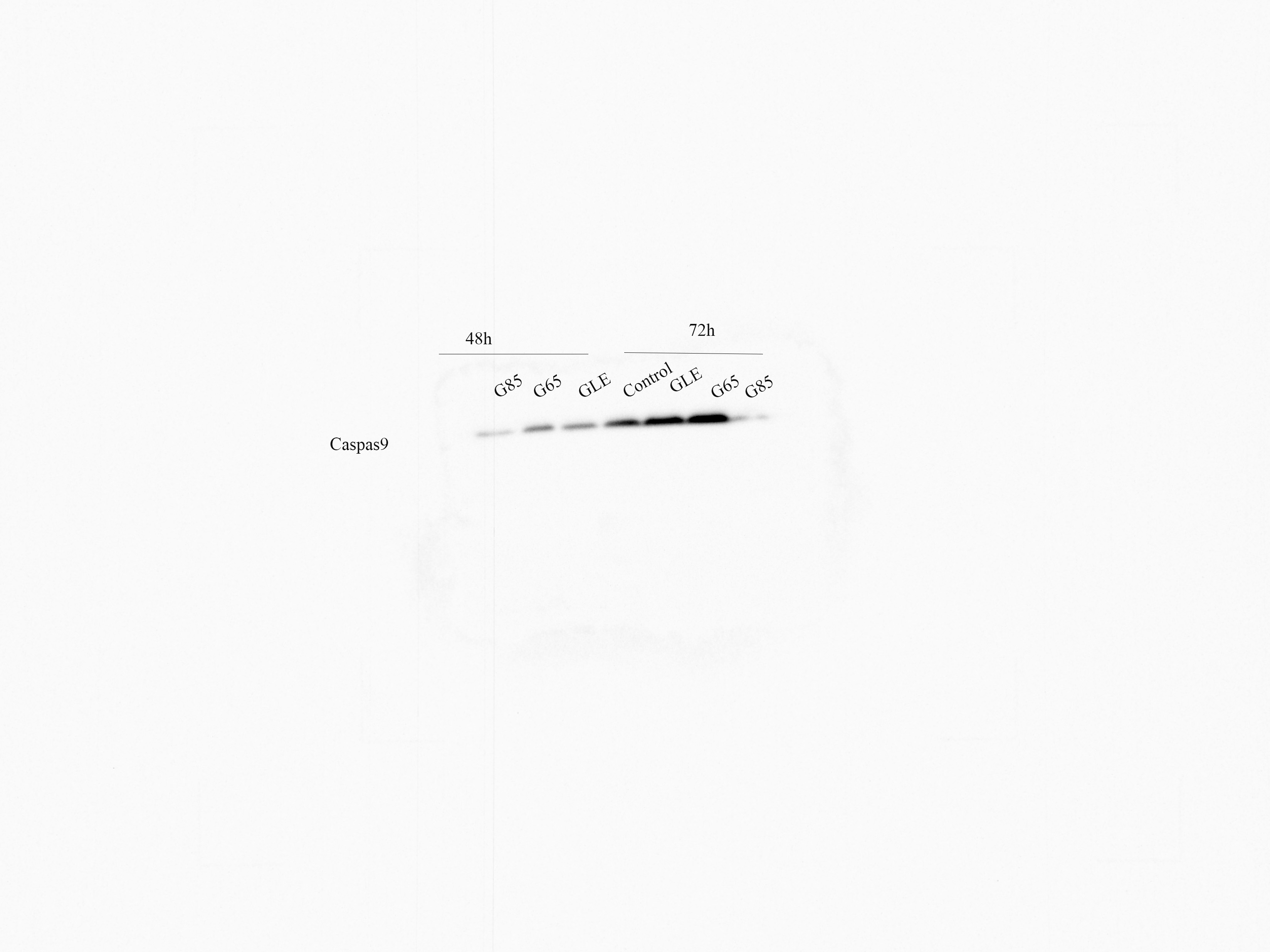

Supplement: Supplementary file 2 [file datasheet2.zip › SK-Hep1 Caspas9.jpg]

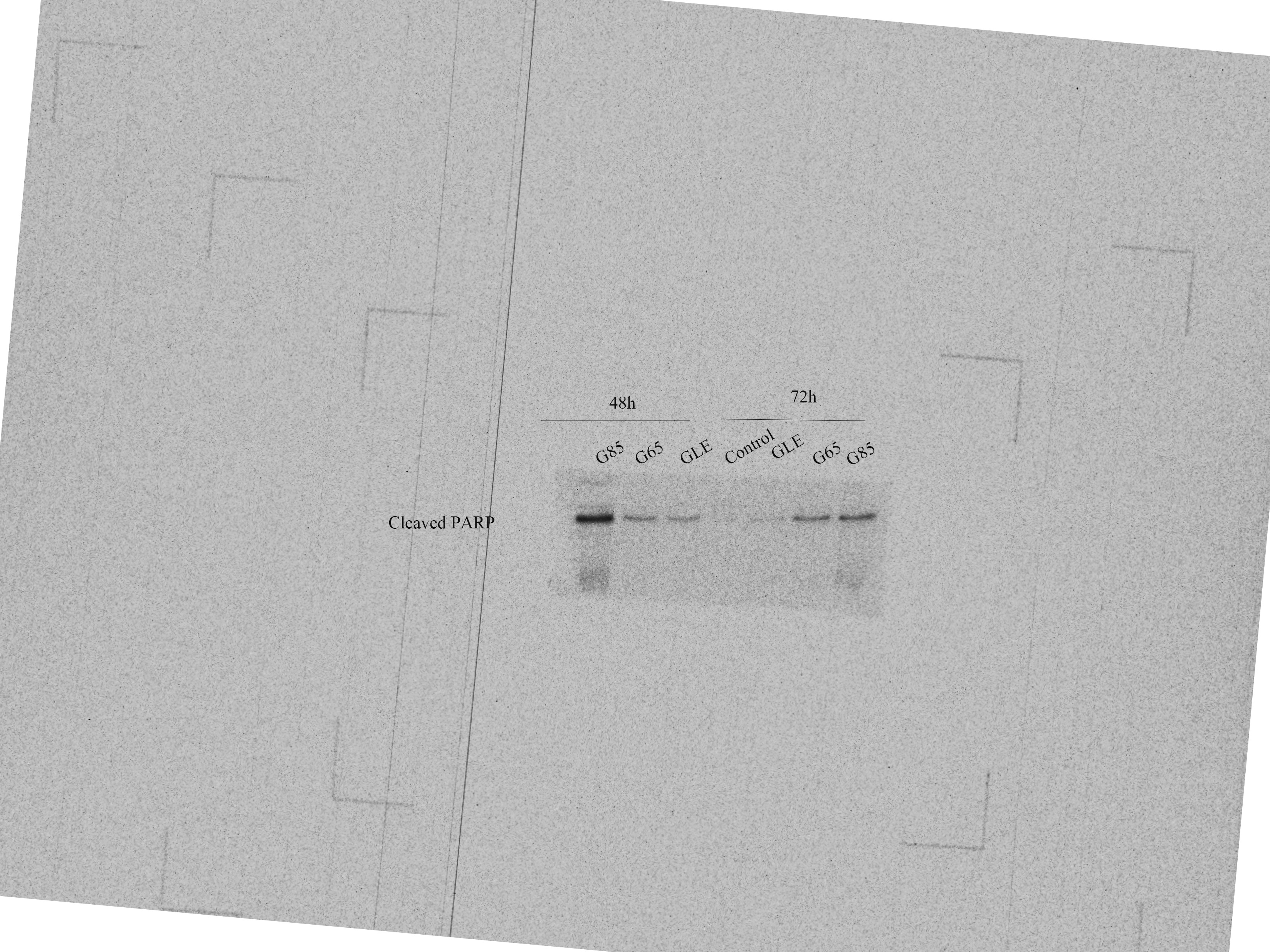

Supplement: Supplementary file 2 [file datasheet2.zip › SK-Hep1 Cleaved PARP.jpg]

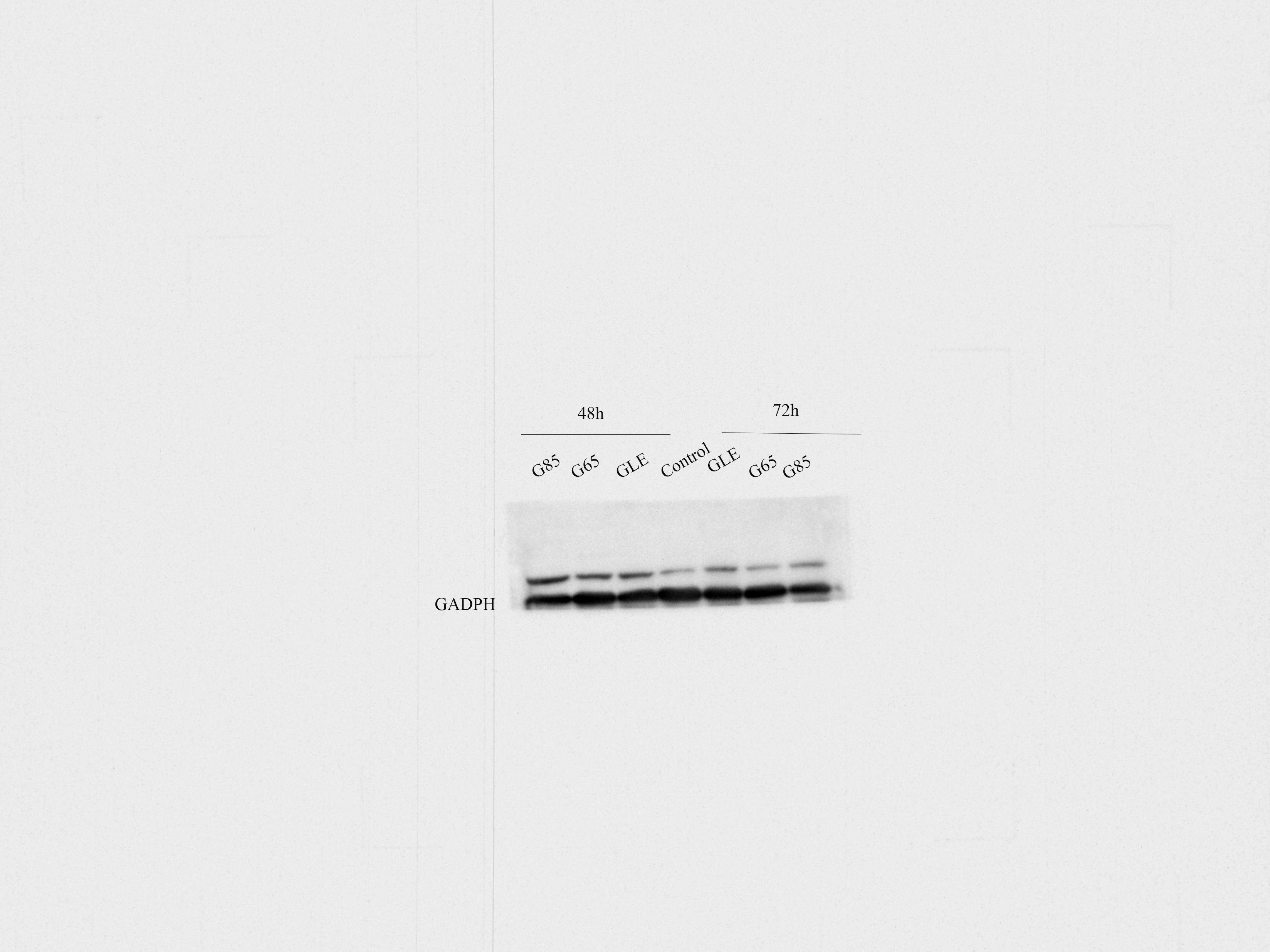

Supplement: Supplementary file 2 [file datasheet2.zip › SK-Hep1 GADPH(apoptosisú⌐.tif]

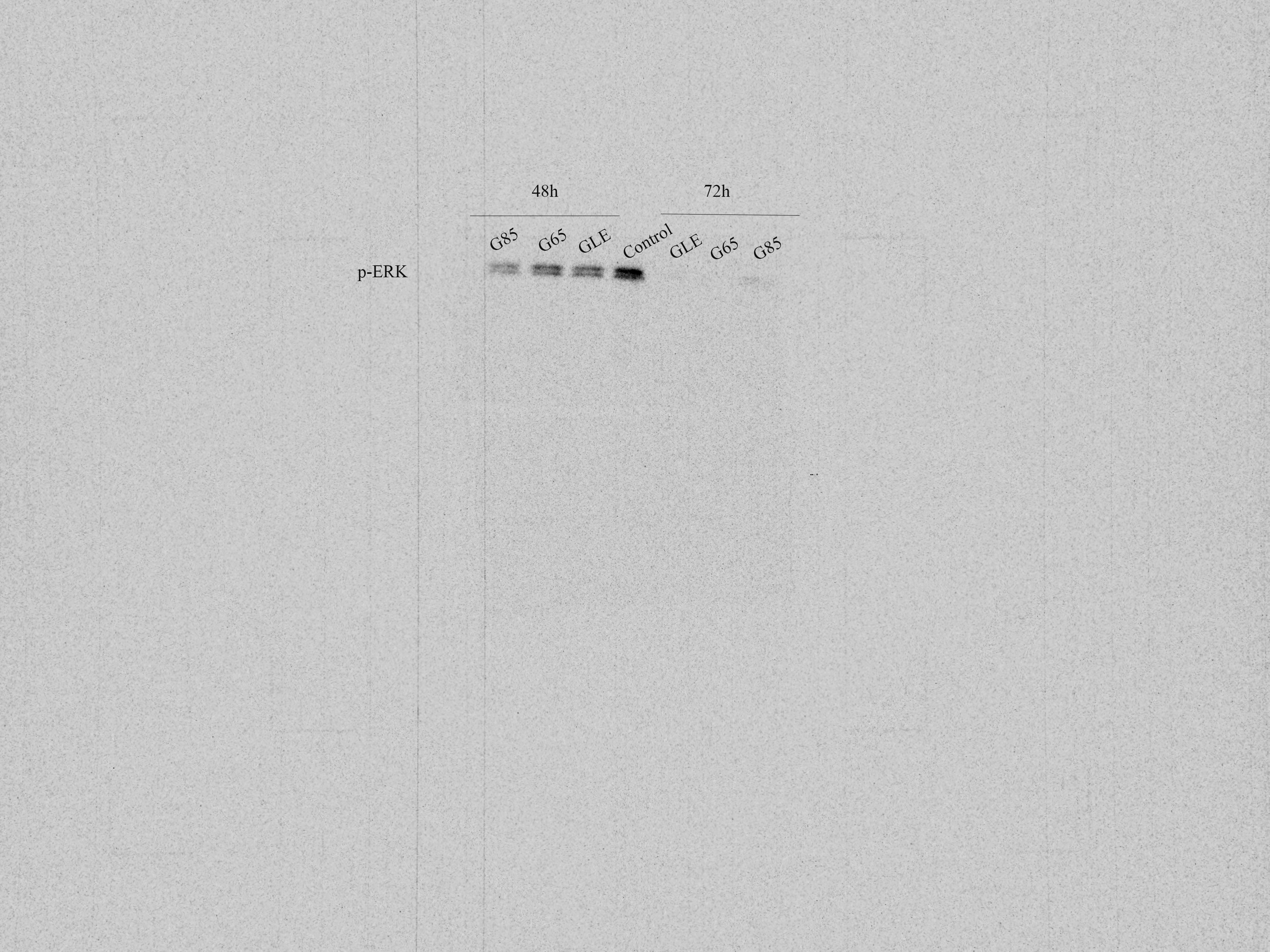

Supplement: Supplementary file 3 [file datasheet3.zip › QGY-7703 p-ERK.jpg]

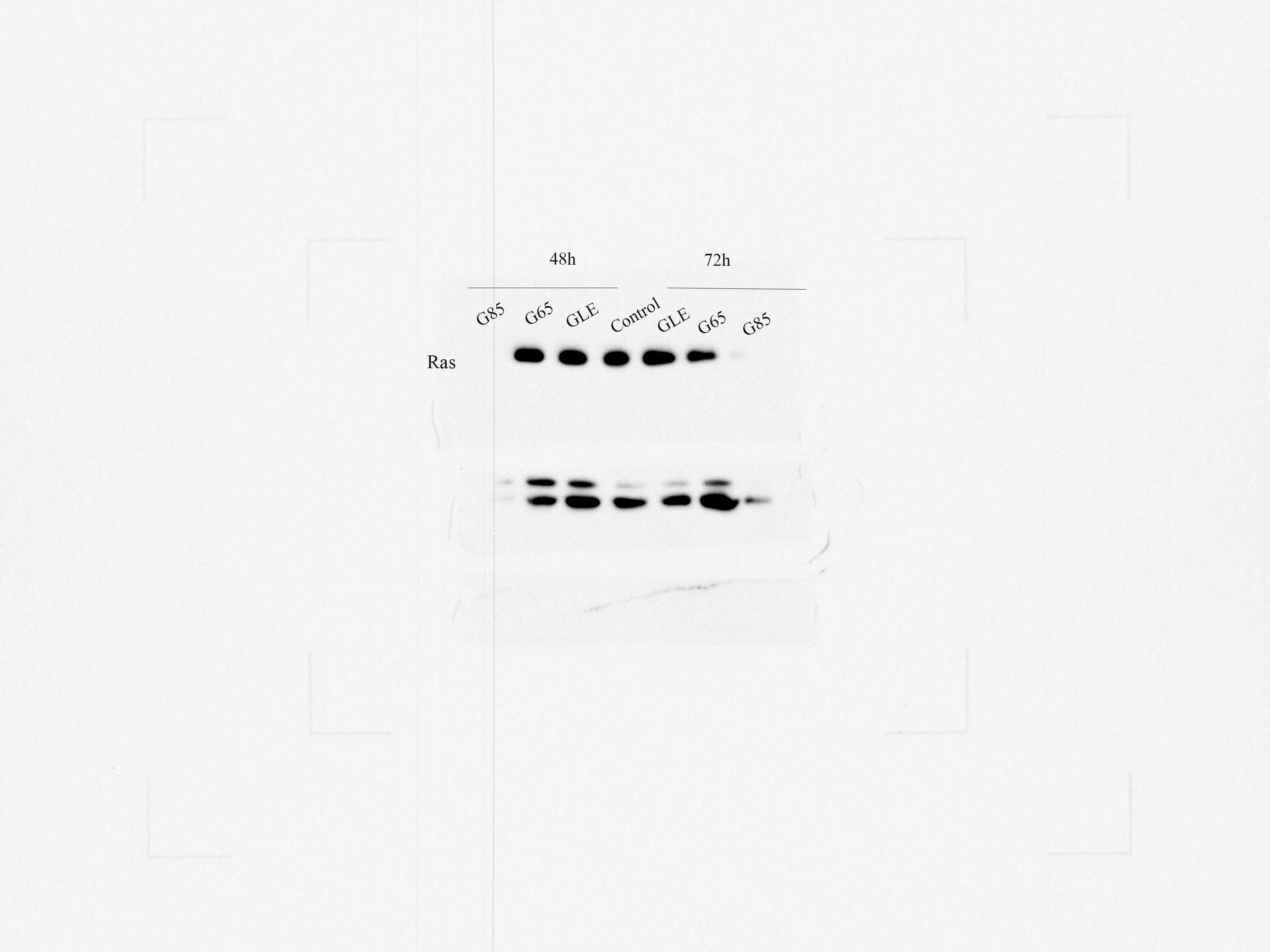

Supplement: Supplementary file 3 [file datasheet3.zip › QGY-7703 Ras (the result of 48h was used. In the result, the original band is rotated 180 degrees.jpg]

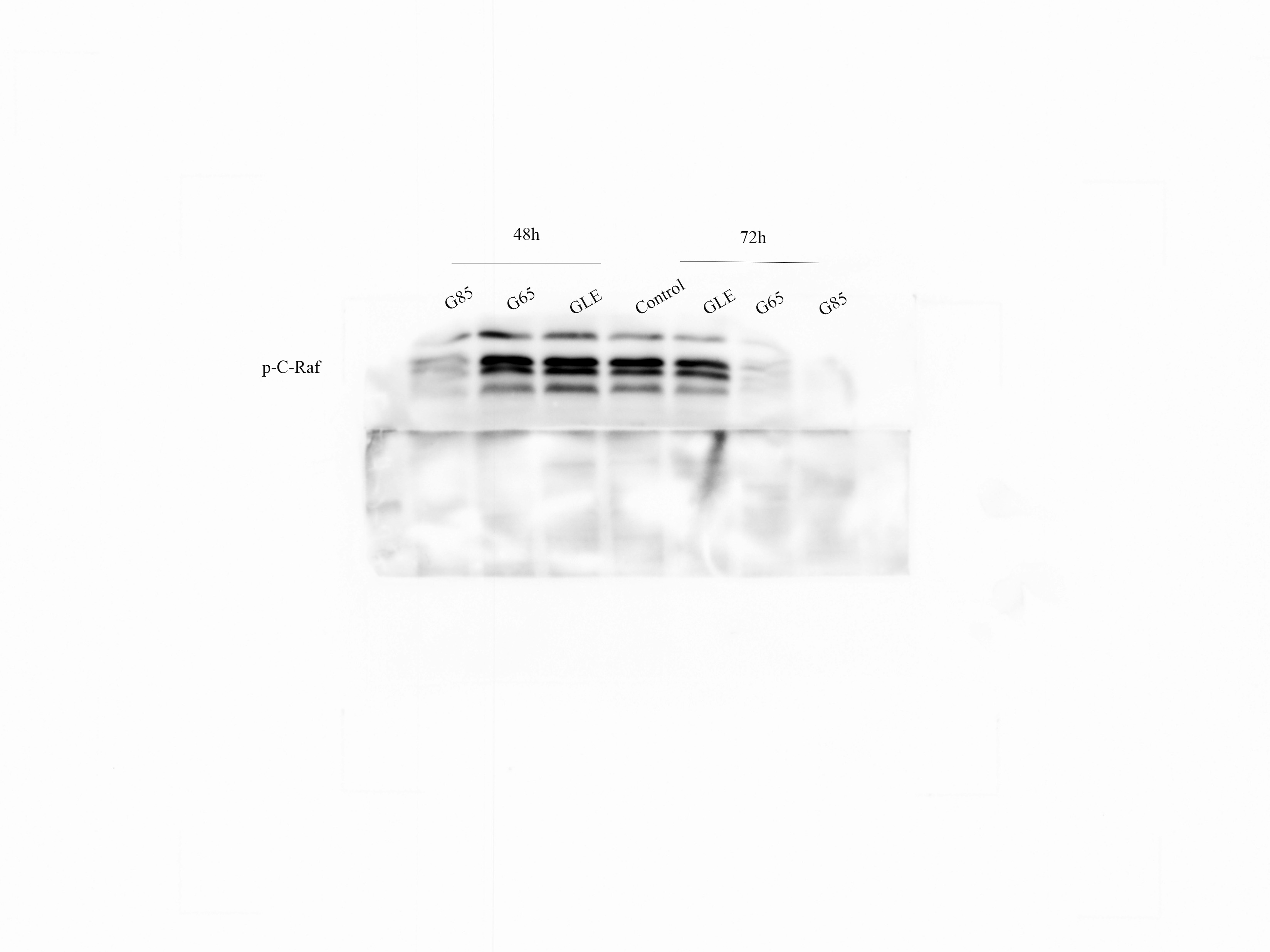

Supplement: Supplementary file 3 [file datasheet3.zip › QGY-7703 p-C-Raf.jpg]

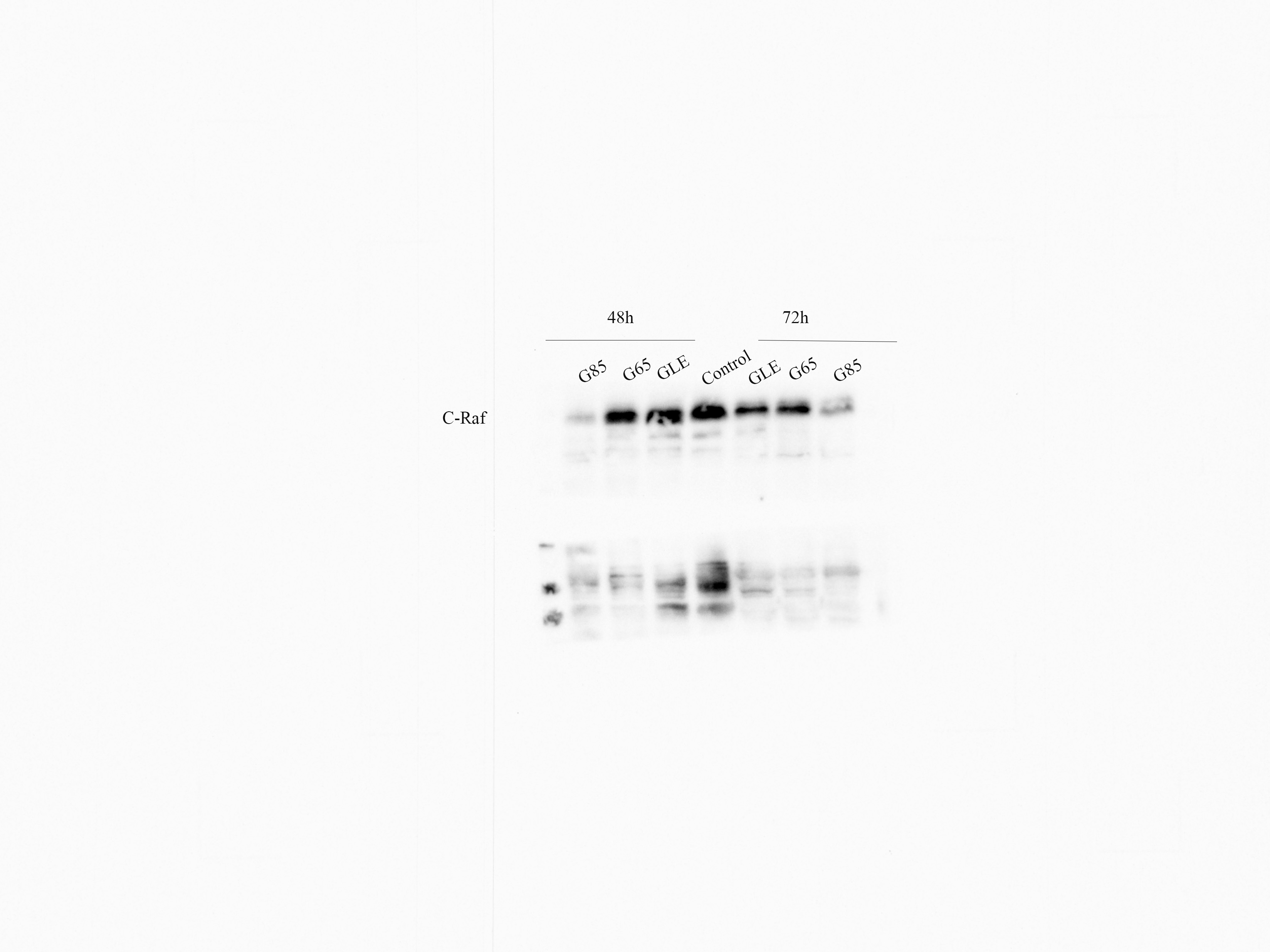

Supplement: Supplementary file 3 [file datasheet3.zip › QGY-7703 C-Raf.jpg]

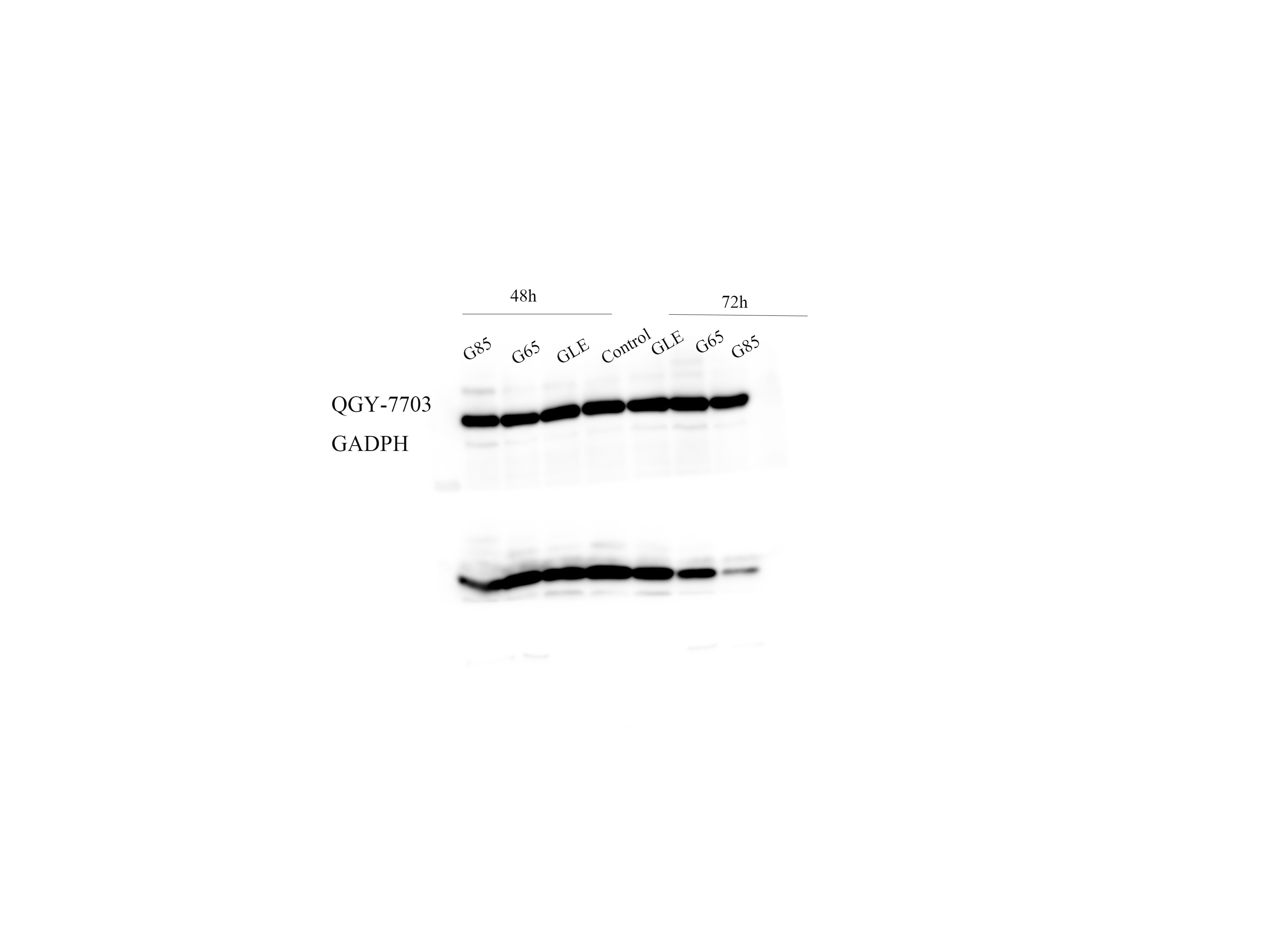

Supplement: Supplementary file 3 [file datasheet3.zip › QGY-7703 GADPH (simultaneous used in MAPK Signaling pathways and apoptosis).jpg]

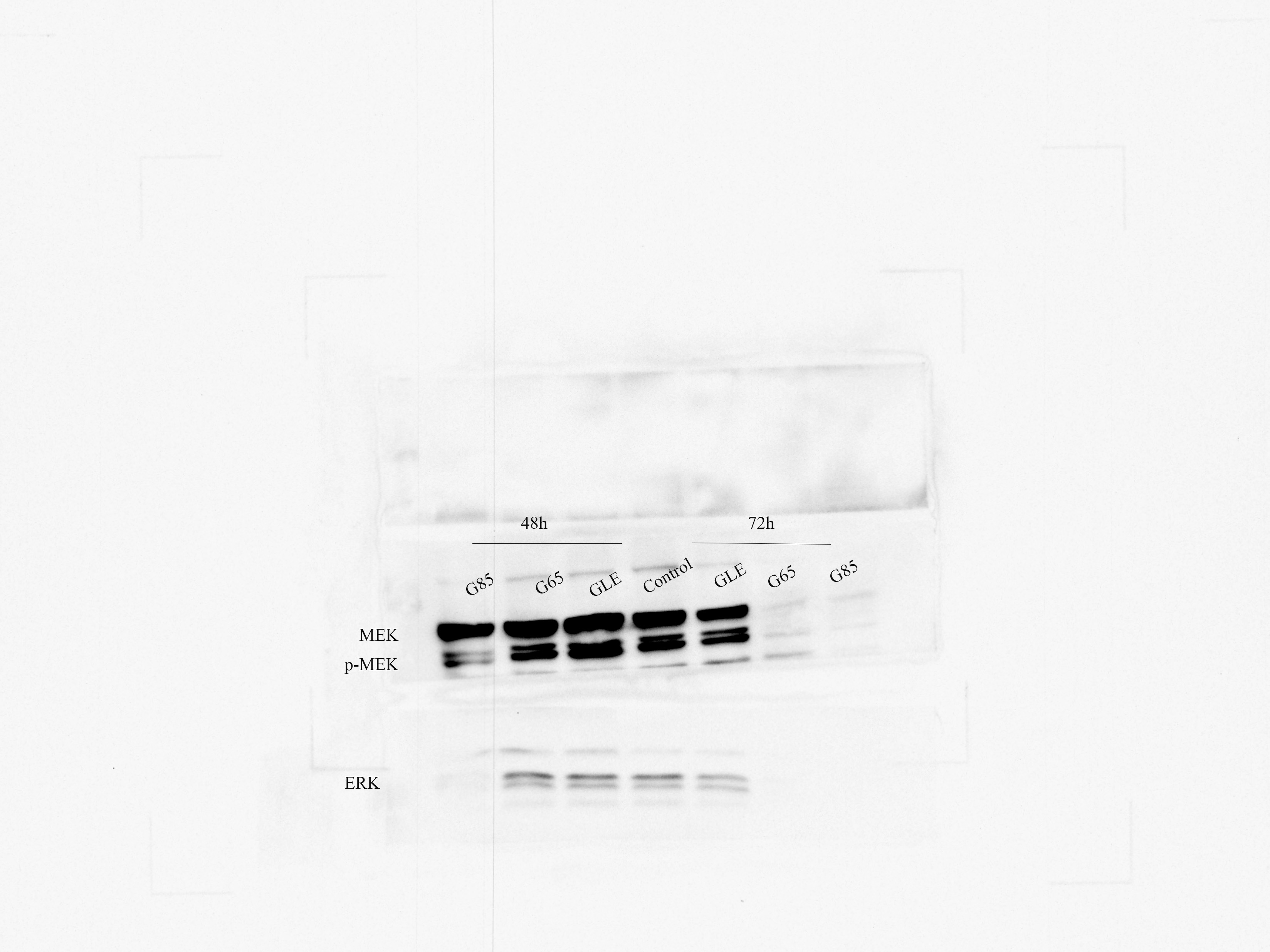

Supplement: Supplementary file 3 [file datasheet3.zip › QGY-7703 MEK and p-MEK(On the same membrane), ERK.jpg]

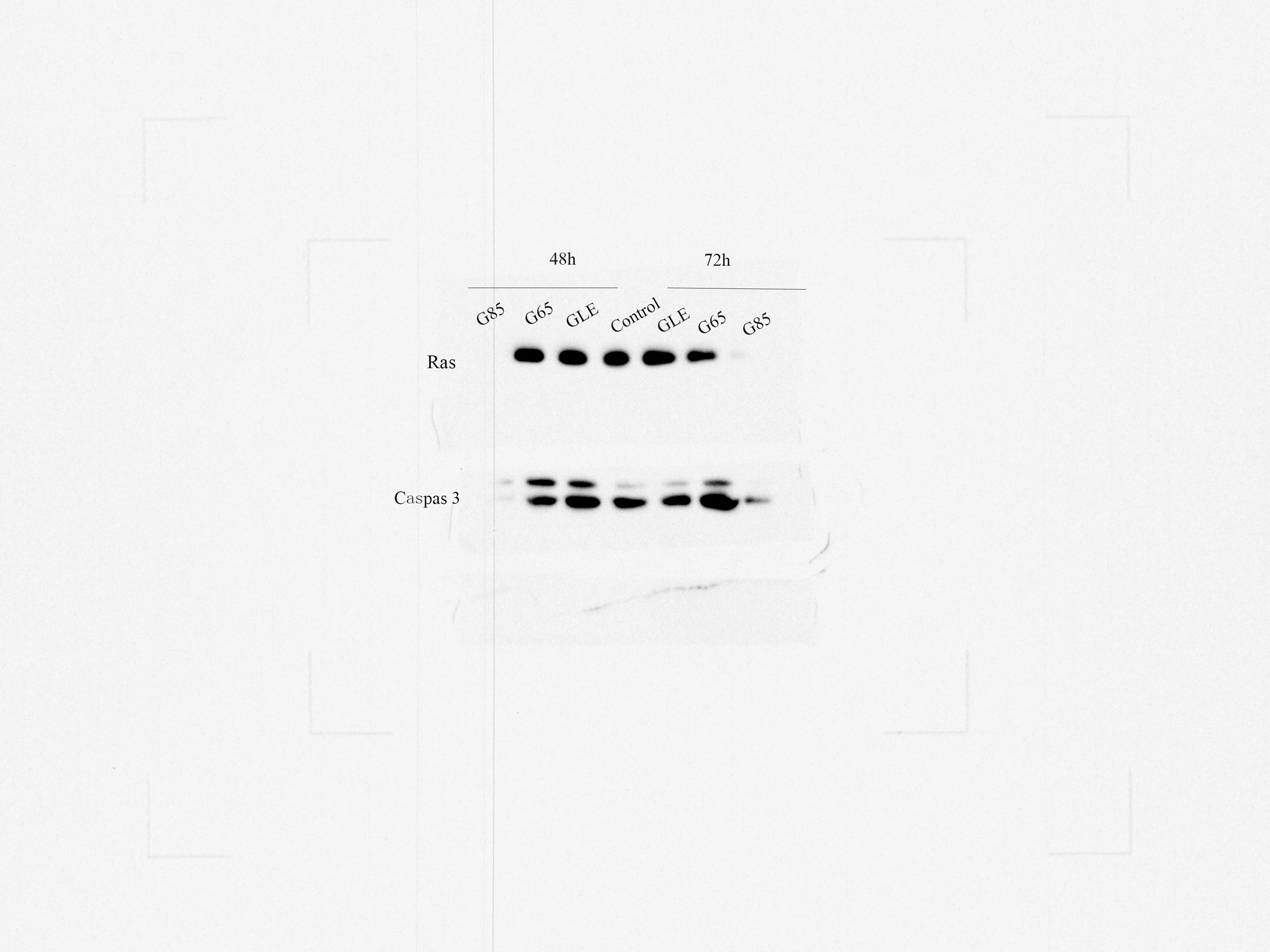

Supplement: Supplementary file 4 [file datasheet4.zip › QGY-7703 Caspas3.jpg]

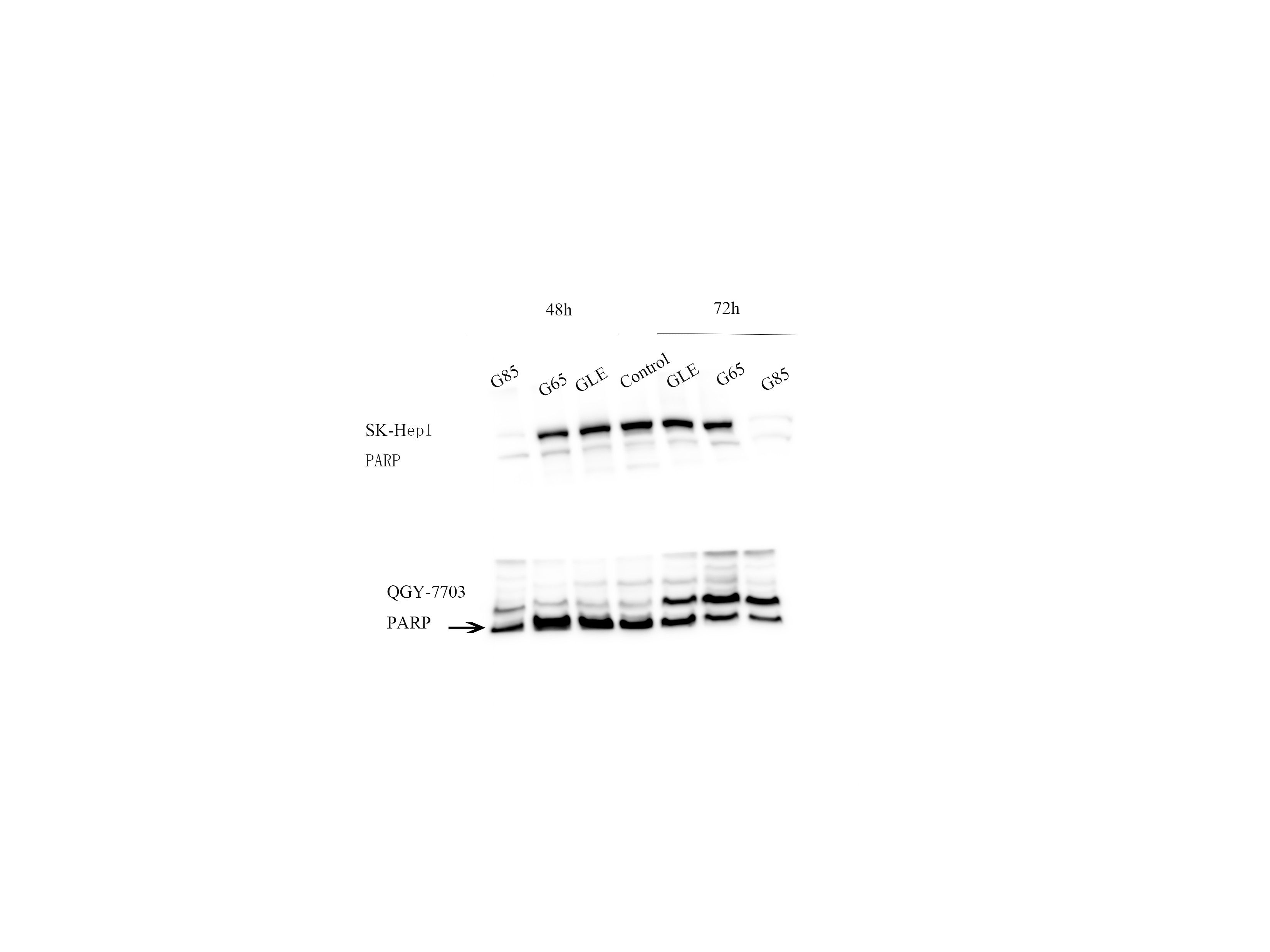

Supplement: Supplementary file 4 [file datasheet4.zip › QGY-7703 PARP.jpg]

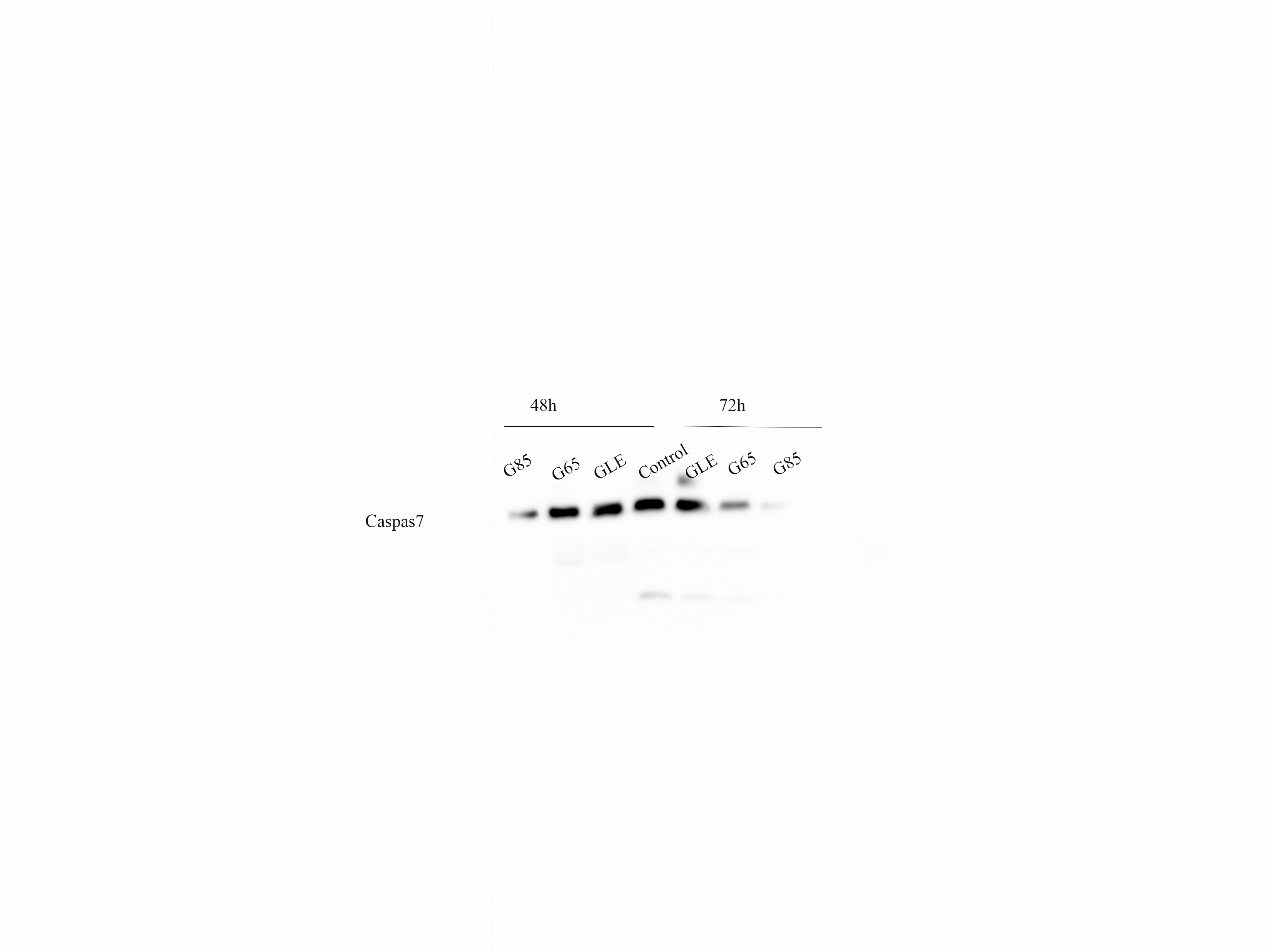

Supplement: Supplementary file 4 [file datasheet4.zip › QGY-7703 Caspas7.jpg]

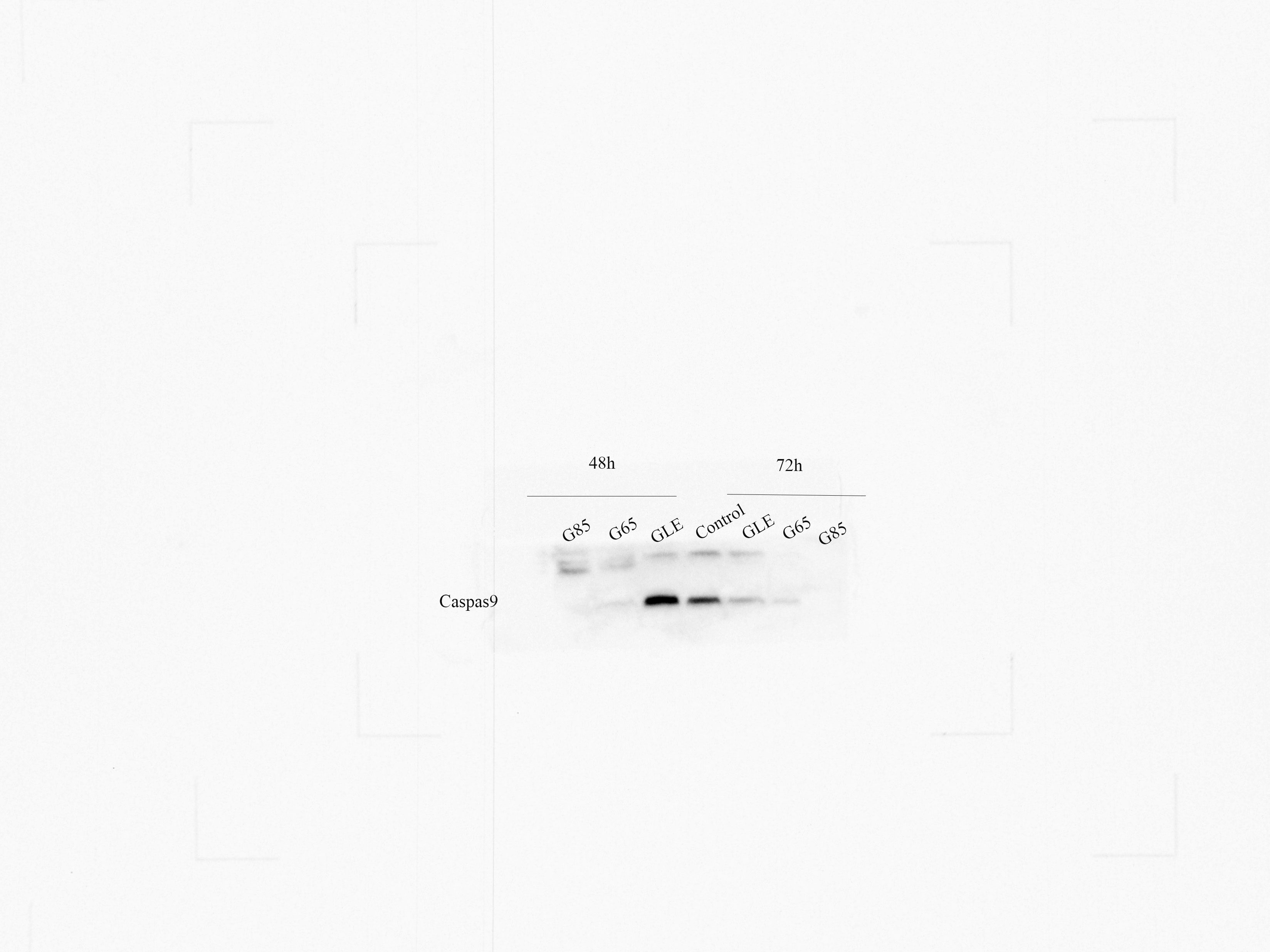

Supplement: Supplementary file 4 [file datasheet4.zip › QGY-7703 Caspas9.jpg]

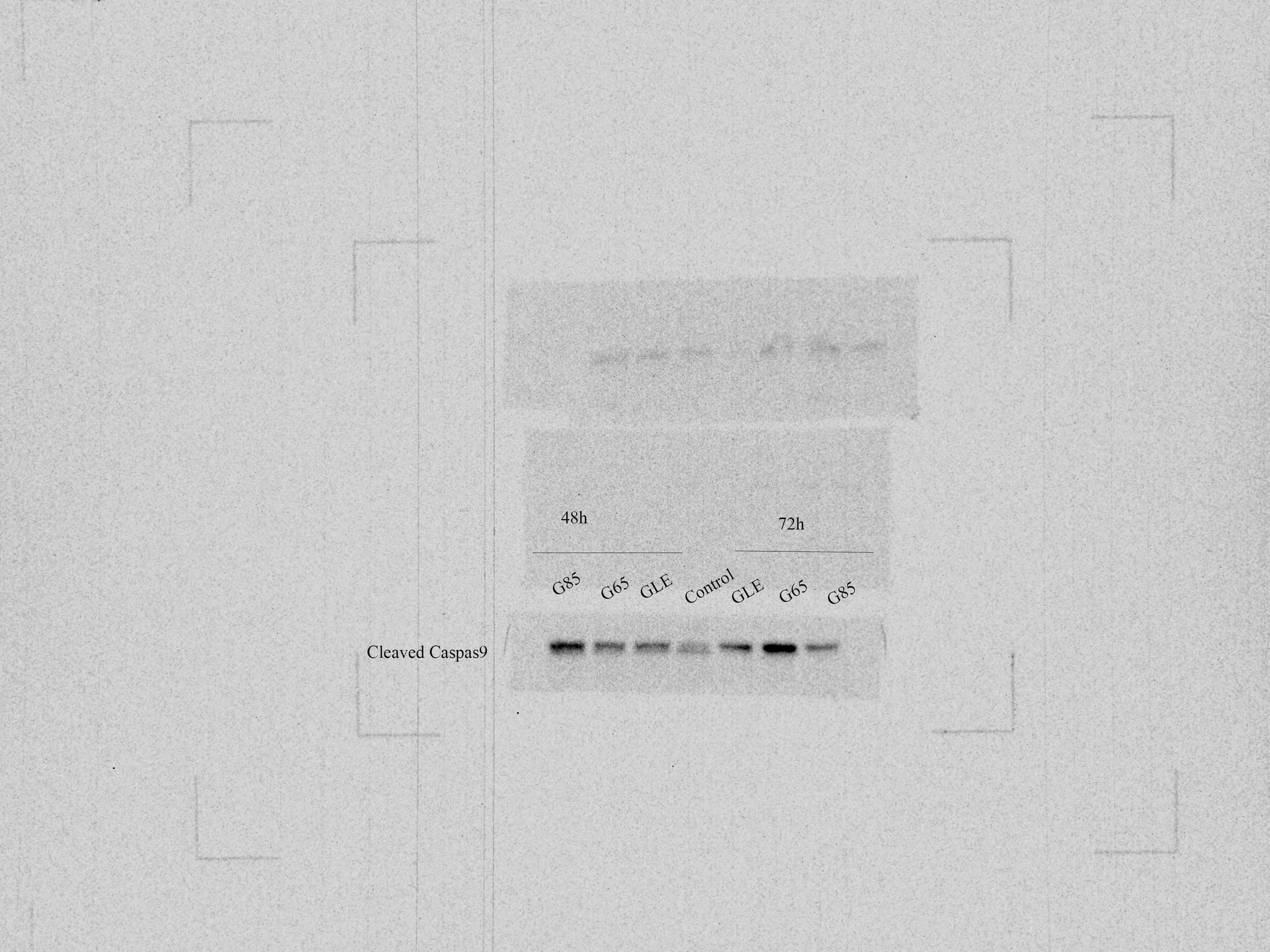

Supplement: Supplementary file 4 [file datasheet4.zip › QGY-7703 Cleaved Caspas9.jpg]

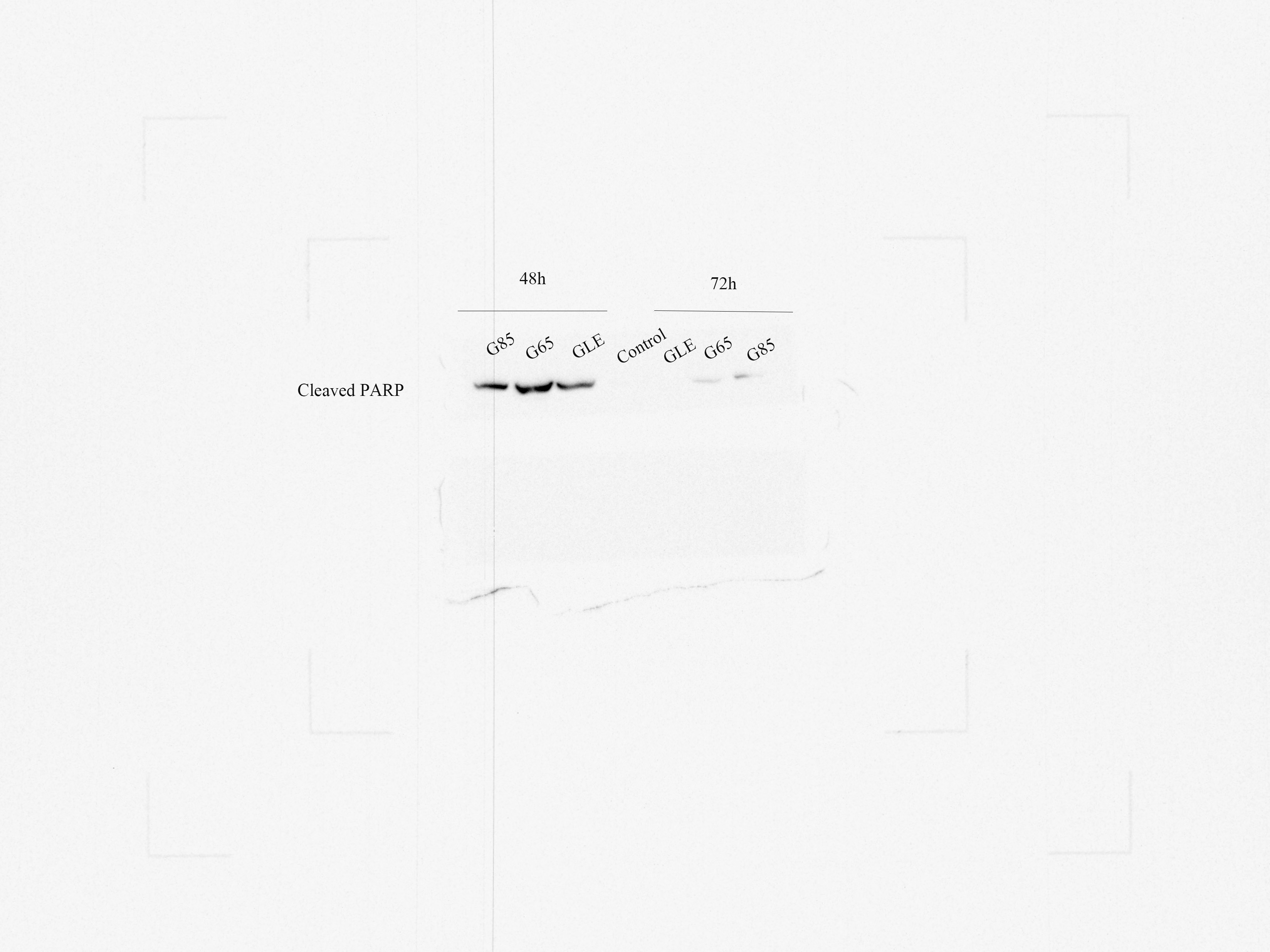

Supplement: Supplementary file 4 [file datasheet4.zip › QGY-7703 Cleaved PARP.jpg]
