# Supplementary material for: High-Pressure Supercritical CO2 Extracts of Ganoderma lucidum Fruiting Body and Their Anti-hepatoma Effect Associated With the Ras/Raf/MEK/ERK Signaling Pathway
Source: Front Pharmacol. 2020 Dec 14;11:602702. doi: 10.3389/fphar.2020.602702 (PMC7768272; doi:10.3389/fphar.2020.602702)
Supplement: Supplementary file 5 [file image1.pdf]

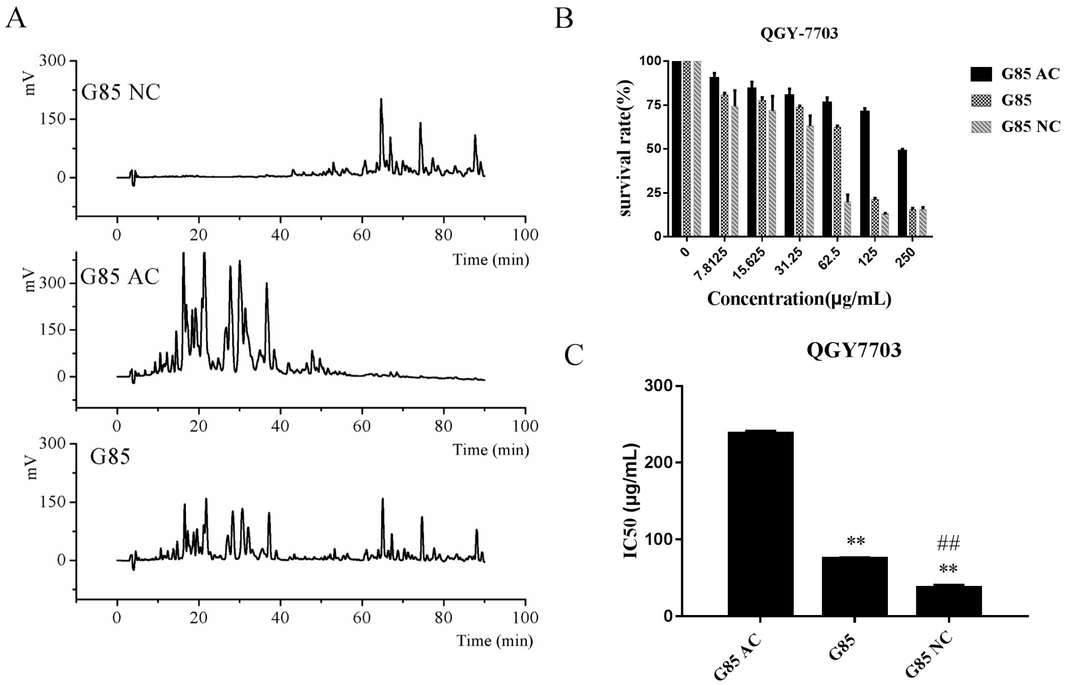

FIGURE S1 | HPLC analysis of G85NC, G85AC and G85 and the comparison of their activity on QGY7703 cells. (A) The HPLC chromatographic profile of G85NC, G85AC and G85. (B) The cytotoxic activity of G85NC, G85AC and G85 on QGY7703 cells. QGY7703 cells were treated for 72 h. Cell viability was measured by the MTT assay. (C) The IC_{50} value of G85NC, G85AC and G85 on QGY7703 cells after exposure of 72 h (n = 3). Data were shown as mean ± SD. $^{**}P<0.01$, vs control. $^{\#\#}P<0.01$, vs G85.
